# Integration of Transcriptome and Methylome Highlights the Roles of Cell Cycle and Hippo Signaling Pathway in Flatfish Sexual Size Dimorphism

**DOI:** 10.3389/fcell.2021.743722

**Published:** 2021-12-02

**Authors:** Na Wang, Qian Yang, Jialin Wang, Rui Shi, Ming Li, Jin Gao, Wenteng Xu, Yingming Yang, Yadong Chen, Songlin Chen

**Affiliations:** ^1^ Key Laboratory for Sustainable Development of Marine Fisheries, Ministry of Agriculture and Rural Affairs, Yellow Sea Fisheries Research Institute, Chinese Academy of Fishery Sciences, Qingdao, China; ^2^ Laboratory for Marine Fisheries Science and Food Production Processes, Qingdao National Laboratory for Marine Science and Technology, Qingdao, China; ^3^ Shandong Key Laboratory of Marine Fisheries Biotechnology and Genetic Breeding, Qingdao, China; ^4^ College of Fisheries and Life Science, Shanghai Ocean University, Shanghai, China; ^5^ Hainan Academy of Ocean and Fisheries Sciences, Haikou, China

**Keywords:** cell cycle, Chinese tongue sole (Cynoglossus semilaevis), hippo signaling pathway, methylome, sexual size dimorphism, transcriptome

## Abstract

Sexual size dimorphism (SSD) is the difference in segments or body size between sexes prevalent in various species. Understanding the genetic architecture of SSD has remained a significant challenge owing to the complexity of growth mechanisms and the sexual influences among species. The Chinese tongue sole (*Cynoglossus semilaevis*), which exhibits a female-biased SSD and sex reversal from female to pseudomale, is an ideal model for exploring SSD mechanism at the molecular level. The present study aimed to integrate transcriptome and methylome analysis to unravel the genetic and epigenetic changes in female, male, and pseudomale *C. semilaevis*. The somatotropic and reproductive tissues (brain, liver, gonad, and muscle) transcriptomes were characterized by RNA-seq technology. Transcriptomic analysis unravelled numerous differentially expressed genes (DEGs) involved in cell growth and death-related pathways. The gonad and muscle methylomes were further employed for screening differentially methylated genes (DMGs). Relatively higher DNA methylation levels were observed in the male and pseudomale individuals. In detail, hypermethylation of the chromosome W was pronounced in the pseudomale group than in the female group. Furthermore, weighted gene co-expression network analysis showed that turquoise and brown modules positively and negatively correlated with the female-biased SSD, respectively. A combined analysis of the module genes and DMGs revealed the female-biased mRNA transcripts and hypomethylated levels in the upstream and downstream regions across the cell cycle-related genes. Moreover, the male and pseudomale-biased gene expression in the hippo signaling pathway were positively correlated with their hypermethylation levels in the gene body. These findings implied that the activation of the cell cycle and the inhibition of the hippo signaling pathway were implicated in *C. semilaevis* female-biased SSD. In addition, the dynamic expression pattern of the epigenetic regulatory factors, including *dnmt1*, *dnmt3a*, *dnmt3b*, and *uhrf1*, among the different sexes correspond with their distinct DNA methylation levels. Herein, we provide valuable clues for understanding female-biased SSD in *C. semilaevis*.

## Introduction

Sexual size dimorphism (SSD) refers to the differences in segment or body size between sexes prevalent in various species including drosophilid flies, mammals, birds, reptiles, arachnida, and fish (Cox et al., 2007; Foellmer and Moya-Larano, 2007; Lindenfors et al., 2007; Székely et al., 2007; Mei and Gui, 2015; Halvorsen et al., 2016; McLean et al., 2018). Understanding the genetic architecture of SSD is a major challenge considering the complexity of the growth mechanisms and the sexual influences. In Drosophila, the *diminutive* (*my*c) gene located on the X-chromosome is crucial in the determination of SSD by contributing to the activation of a female-specific gene *transformer* (*tra*) (Mathews et al., 2017). In primates, the sexual brain size dimorphism is regulated by estrogen, suppressing the promoter activities of microcephaly genes (Shi et al., 2015). Similarly, the growth rate in reptiles is influenced by testosterone levels and environmental factors such as temperature and precipitation (Stillwell and Fox, 2007; Cox et al., 2009; Michael et al., 2014; Agha et al., 2018).

In fish species, a female-biased or a male-biased SSD causes a growth disadvantage within a single-sex, subsequently confining the sustainable development of aquaculture. For example, the Chinese tongue sole (*Cynoglossus semilaevis*), a typical female heterogamete (ZW/ZZ) flatfish, exhibits a female-biased SSD and sex reversal from female (ZW) to pseudomale (ZW), which significantly increases the proportion of phenotypic males in the aquaculture (Zhou et al., 2005; Chen et al., 2009; Chen et al., 2014). Previous studies of SSD in *C. semilaevis* have investigated transcriptomic patterns in both female and male individuals (Wang et al., 2014; Wang et al., 2016; Wang et al., 2018) and various growth-related genes including *growth hormone (gh)*, *growth hormone receptor (ghr)*, *pituitary adenylate cyclase activating polypeptide (pacap)*, *growth hormone releasing hormone (ghrh)*, *leptin*, *bone morphogenetic factor 6 (bmp6)*, *bmp7*, and *growth arrest and DNA damage inducible gamma (gadd45g)* have also been identified by expression levels analysis (Ji et al., 2011; Ma et al., 2012a; Ma et al., 2012b; Liu W.-J. et al., 2014; Ma et al., 2017; Xu et al., 2018). However, the mechanism of SSD in pseudomale is still unknown.

Despite the female and pseudomale having the same genetic background, they exhibit substantial growth differences, which their epigenetic mechanism could explain. Epigenetic modifications such as DNA methylation play crucial roles in gene expression, histone modification, and maintenance of genome stability (Cedar and Bergman, 2009; Cedar and Bergman, 2012). DNA methylation regulates plant species’ growth and development processes (Bartels et al., 2018) and the body weight of male and female mammals (Gonzalez-Nahm et al., 2018). In Nile tilapia (*Oreochromis niloticus*), the epigenetic status of *gh* promoter is negatively correlated with male growth superiority (Zhong et al., 2014). However, in *C. semilaevis*, the relationship between DNA methylation and growth trait was studied only in a few genes, including *gh*, *igf1*, *ghr1*, and *pacap* (Zhao et al., 2015; Si et al., 2016; Li et al., 2017). Much of the research on the whole-genome DNA methylation in *C. semilaevis* has focused on its sexual reversal mechanism and regulation of disease resistance pathways (Shao et al., 2014; Xiu et al., 2019). Moreover, whole-genome DNA methylation and its effects on the SSD transcription patterns in *C. semilaevis* remains unknown.

Thus, the present study aimed to explore the female-biased SSD in *C. semilaevis* through whole-genome transcriptomic and weighted gene co-expression network analysis in four organs (brain, liver, gonad, and muscle), DNA methylation analysis in the gonad and muscle. Integrating transcriptome and methylome data in female, male and pseudomale individuals, will provide valuable insights into the crucial pathways and genes involved in female-biased SSD of *C. semilaevis*.

## Results

### Screening of Differentially Expressed Genes From Female, Male, and Pseudomale *C. semilaevis* Transcriptomes

Whole transcriptome analysis of brain, gonad, liver, and muscle were conducted to explore the different growth mechanisms involved in the female, male, and pseudomale *C. semilaevis*. A total of 3.43 × 10^9^ raw reads were obtained from 36 libraries, including FB1-3, MB1-3, PMB1-3, FG1-3, MG1-3, PMG1-3, FL1-3, ML1-3, PML1-3, FM1-3, MM1-3, and PMM1-3 (Supplementary Table S1). After data filtering by removing adapter and low-quality reads, 3.42 × 10^9^ cleaned reads were retrieved (Supplementary Figure S1). The subsequent Pearson correlation coefficients and PCA analysis (Supplementary Figure S2) revealed high correlation coefficients (>0.9222) in the three biological replicates of the gonad, liver, and muscle. The PMB1 sample had a lower correlation (0.6210–0.7007) with other PMB samples and was removed from subsequent analysis.

**FIGURE 1:**
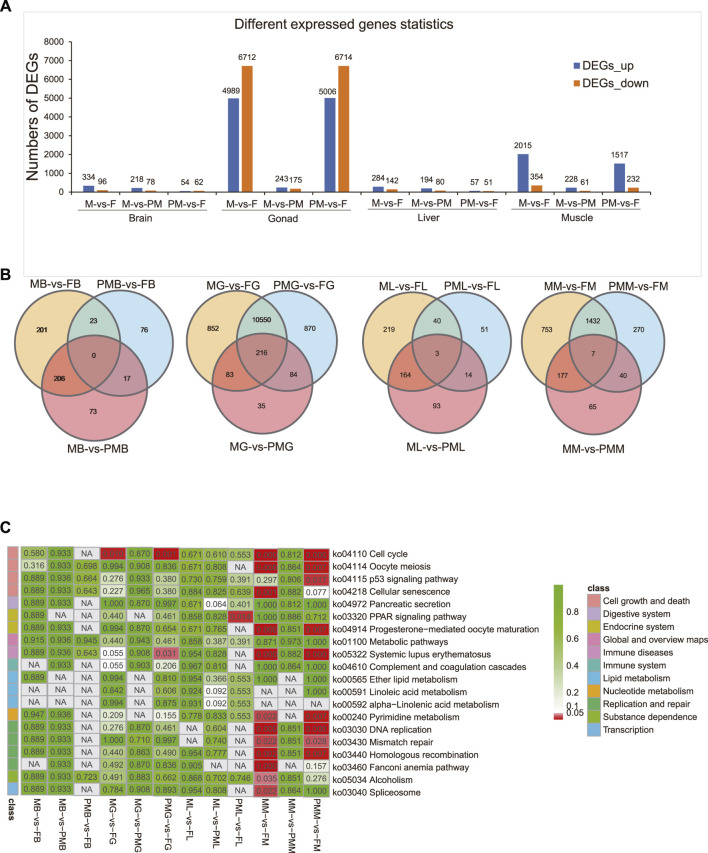
The identification and functional enrichment of DEGs in the whole transcriptome. **(A,B)** The identification of DEGs **(A)** and venn diagram demonstrating DEGs **(B)** from the brain, gonad, liver and muscle of M-vs-F, M-vs-PM, PM-vs-F groups. **(C)** Description of functional KEGG enrichment for DEGs. The color bar means q-value from low (red) to high (green).

After transcripts reconstruction by genome mapping and FPKM calculations, differentially expressed genes (DEGs) were identified (|log2FC| > 1 and q < 0.05) and screened from the M-vs-F, M-vs-PM, and PM-vs-F groups (Figure 1A). The DEGs included: 1) 430, 296, and 116 genes from the brain; 2) 11,701, 418, and 11,720 genes from the gonad; 3) 426, 274, and 108 genes from the liver and 4) 2,369, 289, and 1,749 genes from the muscle. When compared with the female group, the overlapped genes in the male and pseudomale groups from the brain, gonad, liver, and muscle were 23, 10,766, 43, and 1,439, respectively (Figure 1B).

### DEGs Were Affiliated to Many Cell Growth and Death-Related Pathways

The KEGG enrichment analysis (Figure 1C) for DEGs demonstrated that the cell cycle was significantly enriched in the gonad and muscle (q < 0.05). In particular, 104 (71.72%) and 107 (73.79%) DEGs of the cell cycle (145 genes) were classified in the male-vs-female and pseudomale-vs-female gonads, respectively (Supplementary Table S2). Similarly, in the muscle, 53 (36.55%) and 49 (33.79%) DEGs of the cell cycle were screened in the male-female and pseudomale-female groups, respectively. Other cell growth and death-related pathways, including oocyte meiosis, p53 signaling pathway, and cellular senescence, were also significantly enriched in the different sexual muscle tissues. Several replication and repair pathways, DNA replication, mismatch repair, and homologous recombination were also significantly enriched. In the brain and liver, cell growth and death-related pathways, including oocyte meiosis and cell cycle, were identified from the male-vs-female group (*p* < 0.05). However, only fewer than ten DEGs were classified. The GO enrichment analysis of DEGs was shown in Supplementary Figures S3–S6.

### Turquoise and Brown Modules Exhibiting Positive- or Negative-Correlation to Female-Biased SSD Were Identified by Weighted Gene Co-Expression Network Analysis

**FIGURE 2:**
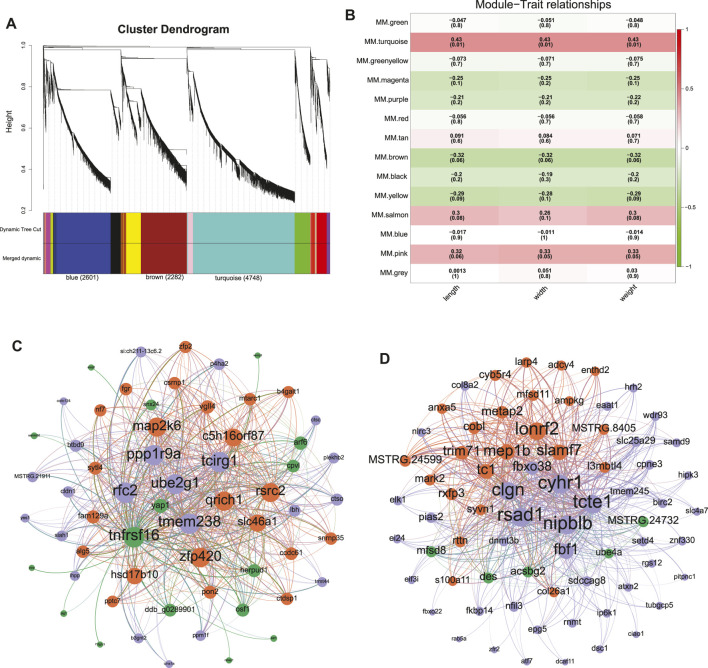
Screening of modules and hub genes by WGCNA. **(A)** The hierarchical clustering of WGCNA. **(B)** The relationship between modules and growth traits. **(C,D)** The gene network constructed by the hub genes in the turquoise module **(C)** and brown module **(D)**.

A total of 13,239 DEGs were obtained by overlapping the DEGs across the four tissues between different sexes to evaluate their correlation with the female-biased SSD in *C. semilaevis*. These DEGs were subsequently submitted for WGCNA analysis, and the gene cluster dendrogram was constructed based on the correlation coefficients of each gene (Figure 2A). Thirteen modules were obtained with module sizes of 95–4,748. The subsequent calculation of module correlation coefficient and sample growth trait (shown in Supplementary Table S3) identified two modules as the most positive and most negative growth trait-related modules (turquoise and brown, respectively in Figure 2B). The high expression levels of genes in the turquoise and brown modules were observed in the female gonad, male and pseudomale gonad, respectively (Supplementary Figure S7).

Genes in the turquoise module were significantly expressed in the following GO terms: catalytic activity, nucleic acid binding, transferase activity, metabolic processes, chromosome organization, RNA processing, and cell cycle (*p* < 0.05) (Supplementary Figure S8). In the brown module, GO terms for cytoplasmic dynein complex, cytoskeleton, dynein complex, and spindle microtubule were significantly enriched (q < 0.05) (Supplementary Figure S9).

**FIGURE 3:**
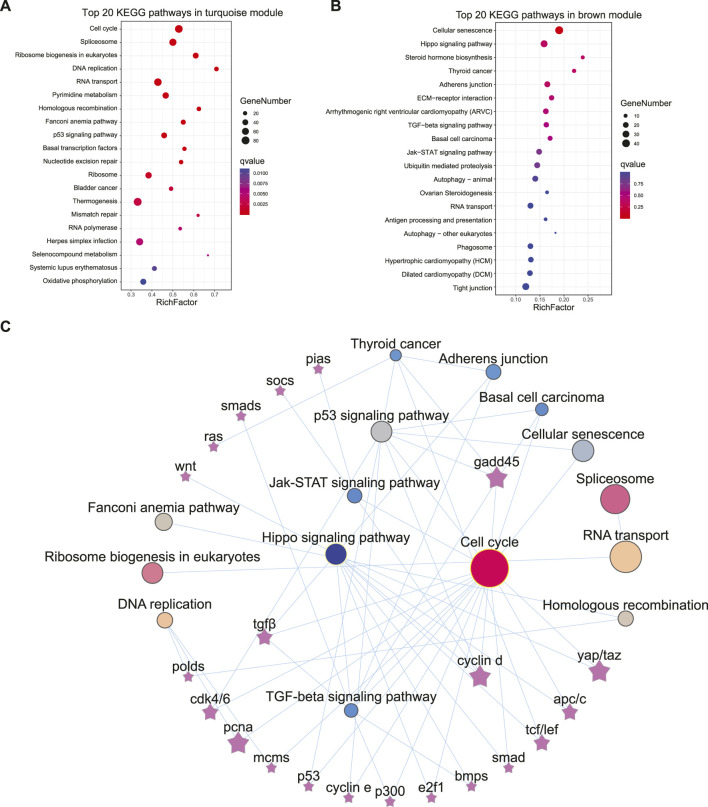
The KEGG enrichment for turquoise and brown modules and the relationship among pathways and genes. **(A,B)** The top 20 enriched KEGG pathways in the turquoise module **(A)** and brown module **(B)**. **(C)** The relationship among the enriched pathways and key genes.

**FIGURE 4:**
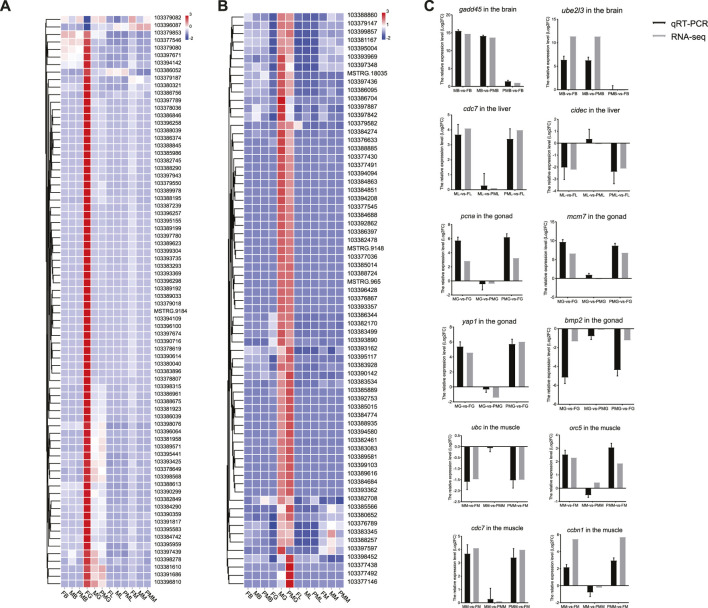
The heatmap of DEGs from the cell cycle, cellular senescence and hippo signaling pathways. **(A,B)** The heatmap of gene expression patterns for cell cycle pathway **(A)**, cellular senescence and hippo signaling pathways **(B)**. **(C)** The qPCR validation of transcriptome gene expression. The color bar means expression levels from low (blue) to high (red).

KEGG enrichment was subsequently employed to reveal the possible functions of these two modules. In the turquoise module, 31 pathways, including cell cycle, spliceosome, DNA replication, etc., were significantly enriched (q < 0.001, Figure 3A). Additionally, 76 DEGs in the cell cycle (Figure 4A) and 65 DEGs in the spliceosome exhibited a female-biased mRNA expression in the gonad. In the brown module, cellular senescence, signal transduction related pathways-Hippo signaling pathway, TGF-beta signaling pathway, and Jak-STAT signaling pathway were significantly enriched (*p* < 0.05) (Figure 3B). A total of 99 DEGs from cellular senescence and hippo signaling pathway displayed male- and pseudomale-biased mRNA expression in the gonad (Figure 4B). The relationship between these pathways revealed that the cell cycle and hippo signaling pathways exhibited the closest interaction with other pathways. Meanwhile, crucial genes-*gadd45*, *cyclin D (ccnd)*, *proliferating cell nuclear antigen (pcna)*, *yorkie homologue* (*yap1,* also known as *yap*), and *tafazzin (taz)* simultaneously participated in multiple pathways were also noted (Figure 3C).

Moreover, the WGCNA network generated using 60 hub genes in the turquoise module (GS. length and GS. weight > 0.50, MM > 0.90, and Weight > 0.60) revealed that *yap1* shared the closest relationship with other genes (Figure 2C). Likewise, *DNA methyltransferase 3b* (*dnmt3b*) displayed the closest relationship with other genes in the brown network constructed using 71 hub genes (GS. length and GS. weight < −0.35, MM > 0.90, and Weight > 0.48) (Figure 2D).

### Expression Pattern Validation of DEGs From Cell Cycle and Hippo Signaling Pathways

The genes involved in the cell cycle and hippo signaling included *gadd45g*, *cyclin-dependent kinase 7* (*cdc7*), *pcna*, *minichromosome maintenance proteins 7* (*mcm7*), *yap1*, *bmp2*, *origin recognition complex subunit 4* (*orc5*), and *cyclin B1* (*ccnb1*). The other genes contained *ubiquitin-conjugating enzyme E2L3* (*ube2l3*), *cell death activator CIDE-3* (*cidec*), and *ubiquitin C* (*ubc*), which were all selected for expression pattern validation by qPCR (Primer information listed in Supplementary Table S4). Similar expression patterns of DEGs in transcriptomes were observed in the four tissues from three comparison groups (Figure 4C).

### Whole-Genome Bisulfite Sequencing of Gonad and Muscle From Different Sexual Groups

#### Methylation Analysis of Gonad and Muscle From Different Sexual Groups

**FIGURE 5:**
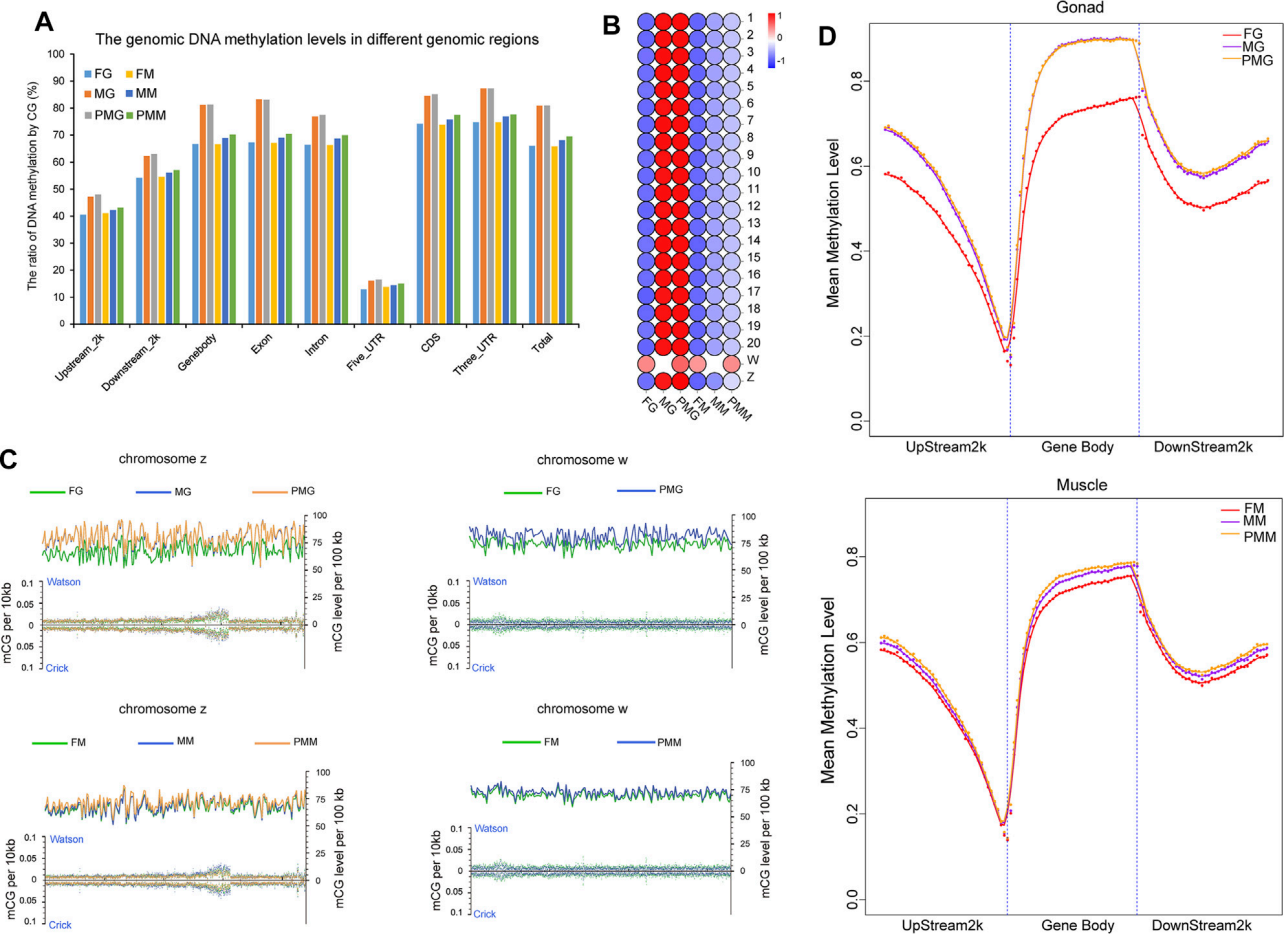
The DNA methylation patterns in three sexual groups by different genomic regions and chromosomes. **(A)** The DNA methylation patterns in different genomic regions of female, male and pseudomale gonads and muscles. **(B)** Illustration of DNA methylation levels in different chromosomes of the six groups. **(C)** Manifestation of methylation status for w and z chromosomes in the different groups. The left *Y*-axis illustrated mCG density in per 10 kb (mCG number/10 kb), and the right *Y*-axis presented mCG density per 100 kb by calculating each CG site. **(D)** The mean methylation levels in the gonad and muscle from three sexual groups. The color bar means methylation levels from low (blue) to high (red).

The gonad and muscle tissues with the highest number of DEGs between individuals of different sex were subjected to WGBS analysis to reveal the possible epigenetic mechanism. A total of 2.08 × 10^9^ reads with the sequencing depth of 27.36× were obtained, and 70.26% mapped to the reference genome (Supplementary Table S5). The sequencing depth and the cumulative distribution of effective sequencing depth by C base proved the uniformity and high quality of the WGBS sequencing (Supplementary Figure S10). The efficiency of bisulfite treatment was >99% after the detection with lambda DNA. The effective C base coverage rates by chromosome, different genomic region, and repeat region were 81.67–97.19%, 91.88–98.54%, and 67.72–95.33%, respectively. The whole genomic methylation analysis revealed that the DNA methylation levels in the male and pseudomale tissues were higher than in the females (Figure 5A). Moreover, the 3′UTR and 5′UTR exhibited the highest and lowest DNA methylation levels, respectively (Figure 5A). In the female gonad and muscle, the highest CG methylation levels was both observed on the W chromosome (Figure 5B). Interestingly, chromosome 17 demonstrated the lowest DNA methylation levels in all examined six groups. A detailed comparison (Figures 5B,C) showed that the total methylation levels of chromosome Z in the gonad and muscle were PM > M > F, while the total methylation levels of chromosome W in these two tissues were F < PM. Furthermore, the dynamic patterns of CG methylation in different genomic regions revealed that the highest and lowest CG methylation levels were observed at the start and end of the gene body, respectively (Figure 5D).

The expression patterns of *dnmt1*, *dnmt3a*, *dnmt3b*, and *ubiquitin like with phd and ring finger domains 1* (*uhrf1*) were illustrated using IGV software (Supplementary Figure S11) to reveal potential roles of these epigenetic regulatory factors in dynamic methylation levels of different sexual groups. Female-biased *dnmt1* and *uhrf1* transcripts were observed in the gonad and muscle tissues. Additionally, the expression levels of *dnmt3b* and *dnmt3a* were F < M < PM in the gonad and muscle tissues.

**FIGURE 6:**
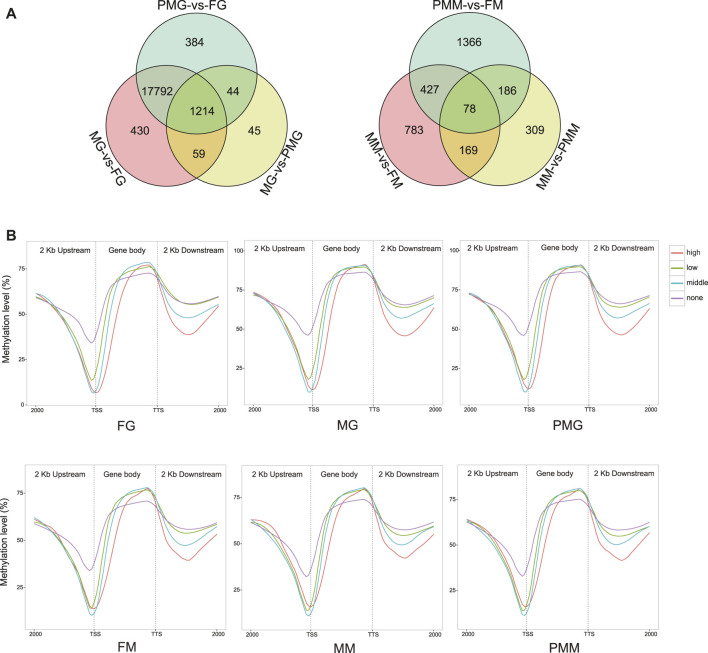
The description of DMGs in the methylome and their linkage analysis within the samples. **(A)** The Venn diagram of DMGs in the six comparison groups. **(B)** The relationship between gene expression and DNA methylation of different regions in the samples. Four groups of DEGs with none (fpkm ≤ 1), low (1 < fpkm ≤ 10), middle (10 < fpkm ≤ 100) and high (fpkm > 100) expression were classified and their mean DNA methylation expression level were subsequently calculated in the 2 kb upstream, gene body, and 2 kb downstream regions.

The identification of differentially methylated regions (DMRs) between two samples resulted in: 1) 414,401 gonad and 2,727 muscle DMRs from the male-vs-female group, 2) 398,065 gonad and 3,949 muscle DMRs from the pseudomale-vs-female group, and 3) 2,872 gonad and 1,293 muscle DMRs from the male-vs-pseudomale group (Supplementary Figure S12A). Further annotation for DMRs revealed 19,968 and 3,318 differentially methylated genes (DMGs) from the gonad and muscle tissues, respectively (Figure 6A). A total of 1,214 gonad and 78 muscle DMGs were separately overlapped in the three comparison groups. The subsequent KEGG enrichment analysis discovered many pathways related to cellular community, signal transduction, and cancers (Supplementary Figure S12B). More than 95% of the genes in the KEGG enriched pathways exhibited differential methylation levels in the gonad comparisons (Supplementary Figures S13A,B). Meanwhile, less than 20% of the genes in the annotation pathway were differentially methylated in the muscle tissues (Supplementary Figures S13C,D).

#### Linkage Analysis of Gene Expression and DNA Methylation Within the Samples and Among the Groups

Four groups of DEGs with no expression (fpkm ≤ 1), low expression (1 < fpkm ≤ 10), moderate expression (10 < fpkm ≤ 100), and high expression (fpkm > 100) were classified. Their mean DNA methylation levels were subsequently calculated in the gene body, upstream and downstream regions. The results revealed negative correlations within 2 kb upstream and 2 kb downstream regions. While, the negative relationship gradually turned positive in the whole gene body region (Figure 6B).

The Spearman’s correlation coefficients between DNA methylation and gene expression within the samples were shown in Supplementary Figure S14. A negative correlation was observed in ±2 kb flanking regions and the gene body.

Common genes between DEGs and DMGs were screened, and their expression patterns were subjected to cluster and enrichment analysis to uncover the contribution of differential DNA methylations to transcript regulation. Results showed that 86.21, 7.89, and 85.83% DEGs in the MG-vs-FG, MG-vs-PMG, and PMG-vs-FG displayed differential DNA methylation (Supplementary Figure S15). Meanwhile, only 6.59, 9.69, and 7.55% DEGs from the MM-vs-FM, MM-vs-PMM and PMM-vs-FM were differentially methylated. In total, 11,087 and 278 overlapped genes were separately screened from the gonad and muscle (Supplementary Figure S15). Subsequently, negative and positive pairs were separately screened from M-vs-F, PM-vs-F, and M-vs-PM groups (Supplementary Figure S15B).

#### Nine-Quadrant Diagram for Module Genes

**FIGURE 7:**
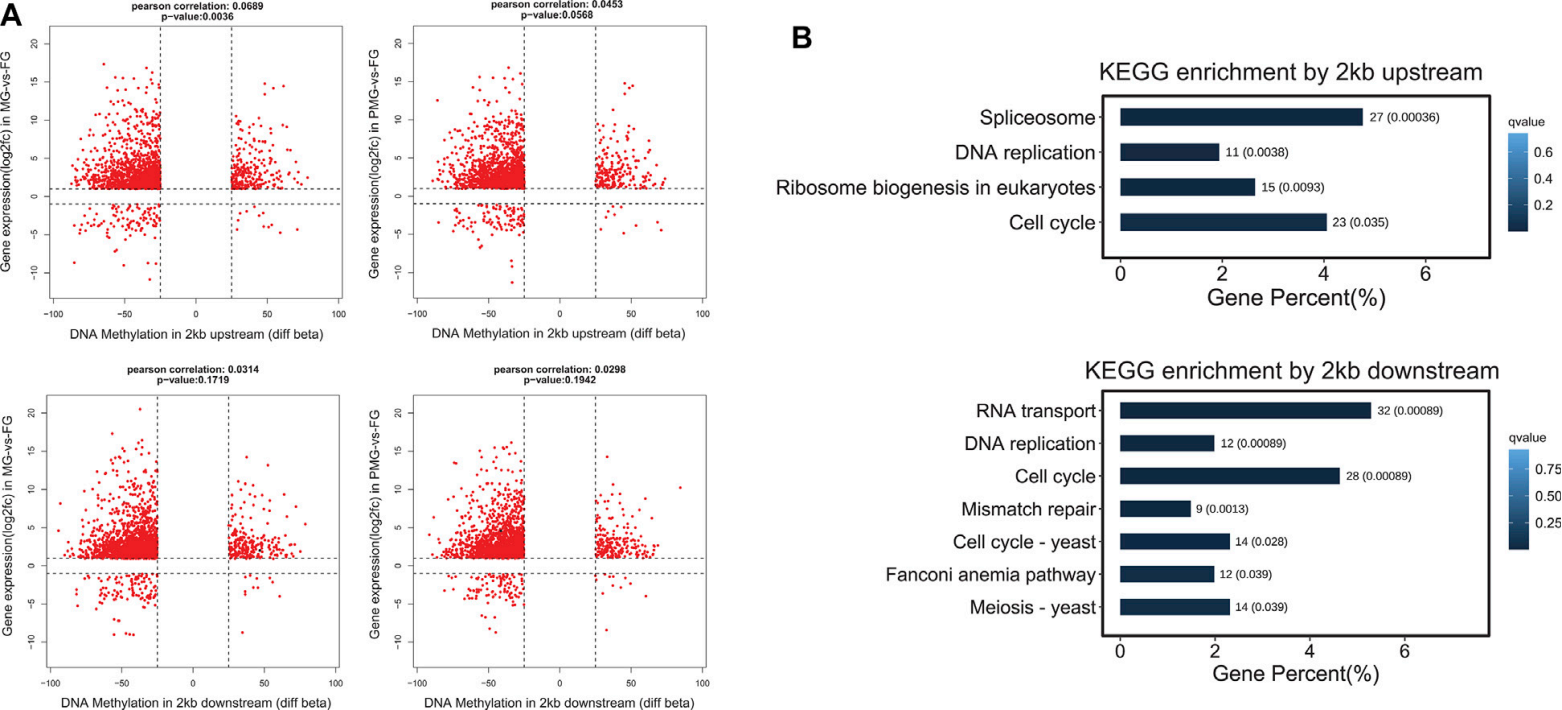
The nine quadrant diagram for turquoise module genes by combining transcripts and DNA methylation levels, and the functional classification. **(A)** The nine quadrant diagram for turquoise module genes and DMGs from 2 kb upstream and 2 kb downstream regions in MG-vs-FG and PMG-vs-FG. **(B)** The functional classification of quadrant 1 **(upper left)** genes by 2 kb upstream and 2 kb downstream.

The DEGs from turquoise and brown modules were separately employed for quadrant diagram construction to understand the potential correlation between mRNA expression and DNA methylation levels. In the turquoise module, 1,420 genes from the MG-vs-FG group and 1,450 genes from the PMG-vs-FG group displayed upregulated transcripts and 2 kb upstream hypomethylation patterns in the female gonad tissues (Figure 7A, quadrant 1). Moreover, these genes were significantly classified into splicesome, DNA replication, ribosome biogenesis, and cell cycle pathways (q < 0.05) (Figure 7B). By analysis of 2 kb downstream methylation patterns, 1,551 and 1,568 genes exhibited upregulated transcripts and hypomethylated levels in MG-vs-FG and PMG-vs-FG groups. Among these genes enriched pathways, DNA replication and cell cycle stand out again (q < 0.05) (Figure 7B).

**FIGURE 8:**
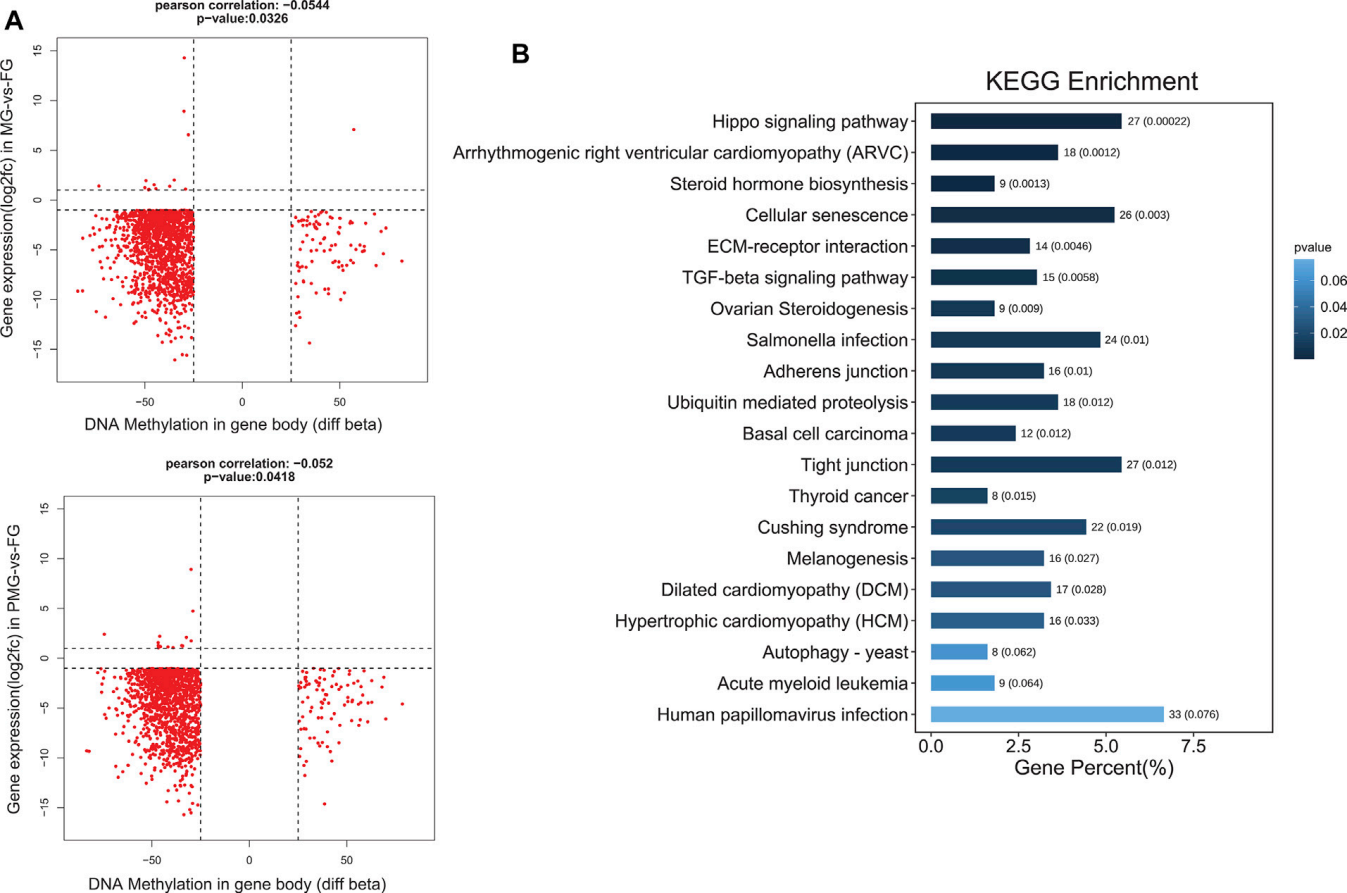
The nine quadrant diagram for brown module genes by combining transcripts and DNA methylation levels, and the functional classification. **(A)** The nine quadrant diagram for brown module genes and DMGs from gene body regions in MG-vs-FG and PMG-vs-FG. **(B)** The functional classification of quadrant 7 **(upper down)** genes by gene body.

In the brown module, 1,435 genes from MG-vs-FG and 1,422 genes from PMG-vs-FG demonstrated downregulated transcripts and gene body hypomethylation patterns in the female gonad (Figure 8A, quadrant 7). Subsequently, these genes were significantly enriched in hippo signaling pathway and cellular senescence (*p* < 0.05) (Figure 8B).

#### The Recognition of Cell Cycle and Hippo Signaling Pathway from the Integration of DMR and DEGs

**FIGURE 9:**
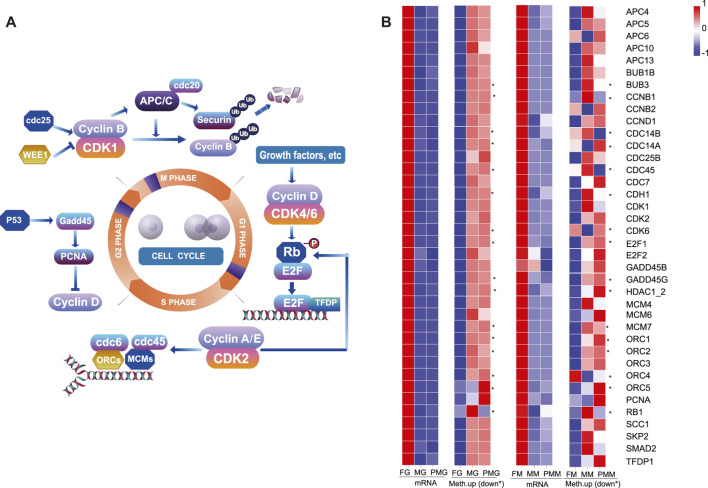
The female-biased expression and hypomethylation of cell cycle genes in the female group. **(A)** Illustration of the cell cycle pathway and important genes. **(B)** The heatmap of 38 cell cycle-related genes in the gonad and muscle by the transcripts FPKM and mean differentially methylation levels in the 2 kb upstream or downstream (*) region. The color bar means expression or methylation levels from low (blue) to high (red).

Based on the nine quadrant diagram results, 38 genes with upregulated transcripts were screened from the cell cycle of the female gonad and muscle tissues. The crucial effectors of cell cycle pathway, *cyclin-dependent kinases* (*cdks*)/*cyclin*, *anaphase-promoting complex* (*apc*)/*cdcs*, *E2F Transcription Factor* (*e2f*), *gadd45*, *pcna*, *orcs*, and *mcms* (Figure 9A), were also involved. Remarkably, their 2 kb upstream and downstream regions demonstrated hypomethylation levels in the female gonad and muscle tissues (Figure 9B).

**FIGURE 10:**
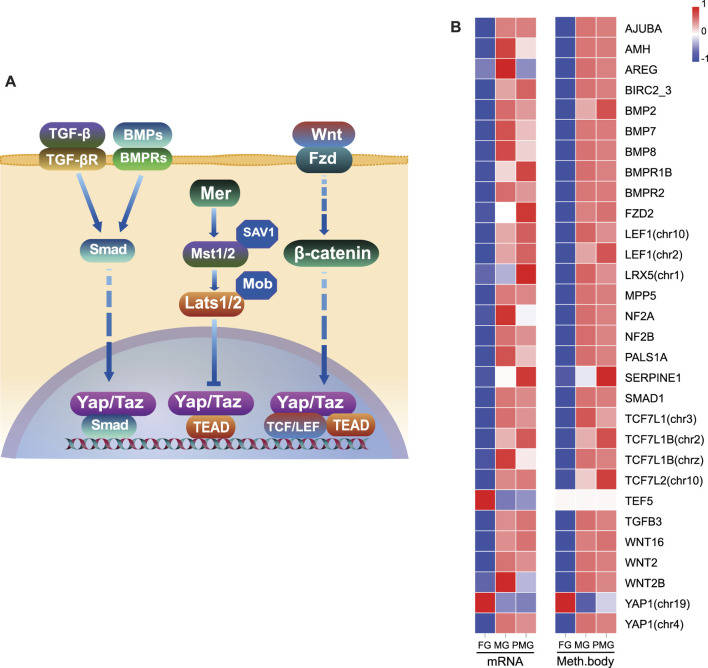
Inhibition of hippo signaling pathway and their gene body hypomethylation in the female gonad. **(A)** Illustration of hippo signaling pathway and essential genes. **(B)** The heatmap of 30 hippo pathway-related genes in the gonad by RNA transcripts FPKM and mean differentially methylation levels in the gene body region. The color bar means expression or methylation levels from low (blue) to high (red).

In the hippo signaling pathway (Figure 10A), 28 genes exhibited downregulated transcripts in the female gonad tissues (Figure 10B), except for the *yap1* (chr19) and *transcriptional Enhancer Factor 5* (*tef5*) (also known as *TEA domain transcription factor 3, tead3*). A total of 30 genes comprised the core hippo signaling factors-*yap1*, *mer* (*nf2a* and *nf2b*), and *tef5*. Additionally, non-canonical hippo signaling pathway-*bmps*, *wnts*, *smad*, and *tcf/lef* (Figure 10A) also exhibited downregulated transcripts in the female gonad. Consistent with WGBS data, the expressions patterns of the hippo pathway-related genes of the gonad tissues positively correlated with DNA methylation in the gene body.

## Discussion

SSD is a common phenomenon in many species and has received much attention on its evolutionary drivers. Although the Rensch’s Rule states that body size variation increases in male-biased SSD and decreases in female-biased SSD (Rensch, 1950), some studies have revealed that many organisms do not conform to this rule (Webb and Freckleton, 2007; Liao et al., 2013; Martin et al., 2017). To date, three hypotheses have been proposed to explain sexual size allometry: evolutionary constraints, natural selection, and sexual selection (Dale et al., 2007; McLean et al., 2018). However, the molecular mechanism of SSD in *C. semilaevis* is complex due to the reversal of female-to-pseudomale and the resembling growth performance between pseudomale and male. Herein, transcriptome and methylome integration was used to assess the female-biased SSD phenomenon in *C. semilaevis*.

### Transcriptome and Methylome Patterns in the Male and Pseudomale Groups

Transcriptome analysis showed many DEGs (10,766 (∼92%) and 1,439 (61–82%) in the gonad and muscle, respectively) overlapped when male and pseudomale were compared with the female group which indicated that males and pseudomales had similar transcriptome patterns, especially in the gonad. Besides, the methylome analysis showed that the DNA methylation levels were higher in the male and pseudomale than in the female groups. Previous studies also reported a similar comparison in the gonad (Shao et al., 2014).

### The Relationship Between Transcriptome and Methylome of *C. semilaevis*

This study found that there were negative correlations in the 2 kb upstream and 2 kb downstream. However, the negative relationship gradually changed to positive in the whole gene body. Studies in mammals and plants have shown that methylation in the promoter and downstream region negatively correlates with the transcript expression (Jones, 1999; Liang et al., 2014). Inversely, the methylation at the TSS inhibits transcript initiation, while the methylation in the gene body can stimulate transcript elongation or affect splicing (Jones, 1999; Jones, 2012). The active transcription is positively associated with gene body methylation in the active X chromosome (Hellman and Chess, 2007).

### The Upregulated mRNA and Hypomethylated of Cell Cycle-Related Genes in the Female Group Might Cause Female-Biased SSD in *C. semilaevis*

Herein, transcriptome analysis revealed the significant female-biased expression of most cell cycle-related genes in the gonad and muscle of *C. semilaevis,* implying their essential roles in the female-biased SSD phenomenon. Undoubtedly, cell cycle is a ubiquitous and complex process that promotes cell genome duplication, growth, and division (Poon, 2016). Cell growth in yeast, bacteria, and plants are usually accompanied with the promotion of cell cycle progression (Goranov et al., 2009; Sablowski and Carnier Dornelas, 2014; Levin and Taheri-Araghi, 2019). Members of *cyclin-cdk* complexes-involved in regulating cell cycle transition through G1, S, G2, M and G0 phases (Schafer, 1998), commonly displayed upregulated transcripts and hypomethylated upstream or downstream region in the female gonad and muscle. Cyclin D is an effective mitogenic sensor that increases expression to convey extracellular signals to the cell cycle via growth factors. The anaphase-promoting complex (APC 4, 5, 6, 10, 13, CDCs) is involved in the proteolysis of cell cycle regulatory proteins (Teixeira and Reed, 2013). E2F-DP complexes (E2F1-2) promote transcription of downstream genes, including cyclin A and E (Poon, 2016). Similarly, other crucial genes-*pcna, gadd45, orc1-5, mcm4, 6, 7,* and *smad2* also exhibited female-biased transcripts and hypomethylated levels at the 2 kb upstream and downstream regions of the gonad and muscle tissues. However, detailed mechanisms of these cell cycle-related genes involved in growth regulation need further exploration.

### The Relationship Between Hippo Signaling and Negative Growth

The hippo pathway-related genes in the negative growth-related brown module suggested their possible roles in the sexual growth difference of *C. semilaevis*. The hippo pathway (conserved in *Drosophila melanogaster* and mammals) has been shown to regulate cell growth, fate decision, organ size, and regeneration (Halder and Johnson, 2011; Ma et al., 2019; Cho and Jiang, 2021). Besides, tissue overgrowth phenotypes in *D. melanogaster* are promoted by the following factors: 1) The mutation of its core kinase components, such as *warts* (*wts*) (Justice et al., 1995; Xu et al., 1995), *hippo* (*hpo*) (Harvey et al., 2003), and *salvador* (*sav*) (Tapon et al., 2002). 2) the loss-of-function of its upstream regulatory factors, including *merlin* (*mer*), *expanded* (*ex*) (Hamaratoglu et al., 2006), *fat* (*ft*) (Cho et al., 2006). 3) Overexpression of its downstream effectors, such as *yorkie* (*yki*) and *scalloped* (*sd*) (Huang et al., 2005; Wu et al., 2008). The overexpression of *yorkie homologue*-*yap* also enhances liver size in mouse by increasing cell proliferation (Dong et al., 2007). Herein, upstream factor, *mer* (*nf2a* and *nf2b*), were downregulated in the female gonad of *C. semilaevis*, and downstream effector-*sd* (*tead3*/*tef5*) were upregulated. Another key downstream factor, *yap1* had two homologues in chromosomes 4 and 19, which separately displayed male- or pseudomale-biased transcripts in the gonad, and female-biased expression levels in the gonad and muscle, indicating the complexity of the hippo pathway involved in fish growth regulation. The integration of transcriptome and methylome data suggested that the inhibition of hippo signaling via the canonical and non-canonical pathways was possibly regulated by hypomethylated patterns in the gene body region, especially the gonad.

### The Relationship Among Cell Cycle, Hippo Signaling, and Other Pathways

The tissue overgrowth phenotype induced by the hippo pathway in Drosophila are commonly accompanied and characterized by the activation of cell cycle regulator *cyclin e* (Harvey et al., 2003). Recent studies in various mammal cells have revealed that cell cycle are associated with the hippo pathway. For instance, *yap* knockdown causes cell cycle arrest at the G0/G1 phase by decreasing the cycle-related protein-Cyclin D1 (Chen et al., 2017). In contrast, *yap* activation promotes cell cycle progression by increasing Cyclin D1 (Mizuno et al., 2012; Xie et al., 2019). Besides *cyclin e* and c*yclin d*, other cell cycle genes, including *cdc6* and *e2f1*, are also downstream targets of *yap* (Kapoor et al., 2014; Kim et al., 2019). Moreover, the cell cycle controlling factor, APC/Cyclosome (APC/C)^cdh1^ E3 ubiquitin ligase complex, can increase YAP/TAZ activities by promoting LATS1/2 kinase degradation (Kim et al., 2019). Herein, the possible downstream targets, including *cyclin d1*, *cyclin e1*, *cyclin e2*, *cdc6*, and *e2f1*, the possible upstream, *apc/c* and *cdh1* genes exhibited female-biased mRNA levels in the gonad of *C*. *semilaevis*.

Additionally, the GH-IGF system, highly conserved in somatotropic axis across various species, including mammals and fish (Baker et al., 1993; Reinecke et al., 2000; Ahmed and Farquharson, 2010), participate in growth regulation via JAK-STAT, PI3K-Akt signaling etc. (Brooks et al., 2014; Ipsa et al., 2019; Rotwein, 2020). IGF1, a crucial factor of the GH-IGF system, can inhibit the hippo pathway via PI3K-Akt signaling, thus activating YAP/TAZ effector to promote cell proliferation (Fan et al., 2013; Fruman et al., 2017; Chen et al., 2018; Thompson, 2020). Herein, *igf1* had upregulated transcripts and hypomethylated patterns in the female gonad of *C.* se*milaevis*. Thus, this phenomenon seems to provide a possible linkage to hippo pathway and classical somatotropic axis regulators.

### The Possible Epigenetic Regulatory Mechanism in *C. semilaevis*

The *de novo* and maintenance of DNA methylation in mammals require DNA methyltransferase enzymes, including DNMT1, DNMT3A, and DNMT3B (Reik et al., 2001; Jones and Liang, 2009). Herein, the pattern of transcripts levels of *dnmt3a* and *dnmt3b* were FG < MG < PMG, indicating a negative correlation with the DNA methylation levels. Besides, the histone variant H2A.Z, strongly antagonistic to DNMTs (Zilberman et al., 2008; Conerly et al., 2010), had two orthologue genes in *C. semilaevis* chromosome w and z with 100% protein homology. Their total expression levels were FG > MG > PMG, while their DNA methylation levels were FG < MG < PMG. Recent studies have revealed that the recruitment of DNMT1 to replication sites occurs via an interaction with PCNA and UHRF1 (Jurkowska and Jeltsch, 2016; Jimenji et al., 2019), which both had the highest expression levels in the female gonad and muscle of *C. semilaevis*. Interestingly, *uhrf1* overexpression can cause DNA hypomethylation, similar to *uhrf1* mutants (Kent et al., 2016). Therefore, its female-biased transcripts could be responsible for the hypomethylation of the gonad and muscle tissues in the female group of *C. semilaevis*. Surprisingly, the highest expression levels of *dnmt1* were detected in the female groups. Therefore, further investigation is required to confirm whether a negative interaction exists between the *dnmt1* and DNA methylation levels.

In conclusion, this study identified many DEGs implicated in various cell growth and death-related pathways through transcriptome analysis of the four organ tissues from three sexual groups. Methylome data revealed that the DNA methylation patterns in the male and pseudomale tissues were higher than in the females. Moreover, the integration of transcriptome and methylome data revealed that cell cycle-related genes were upregulated and hypomethylated in the upstream or downstream regions of the female gonad and muscle. Conversely, the male and pseudomale-biased expression of hippo signaling pathway genes were positively correlated with their hypermethylation levels in the gene body. The activation of the cell cycle and the inhibition of the hippo signaling pathway could be implicated in the *C. semilaevis* female-biased SSD. Additional functional experiments involving essential genes of these pathways and the epigenetic regulatory factors are needed to assess the fish sexual size dimorphism.

## Materials and Methods

### Fish Sampling, Sex Identification, Tissue Collection, and RNA Isolation

In this study, individual fish were anesthetized with MS-222 before the sampling to relieve pain. Four somatotropic and reproductive tissues from the brain, liver, gonad, and muscle were isolated from nine females, nine males, nine pseudomales in triplicate and immediately stored in liquid nitrogen. The fish, each 1.5-years-old *C. semilaevis* species were cultivated in Haiyang Yellow Sea Fisheries Limited Company. The genetic sex identification of the male and the pseudomale was performed using the primers sex-F (CCT​AAA​TGA​TGG​ATG​TAG​ATT​CTG​TC) and sex-R (GAT​CCA​GAG​AAA​ATA​AAC​CCA​GG), described in a previous study (Liu Y. et al., 2014).

Each triplicate sample was pooled per treatment into one sample, generating 36 samples hereafter named FB1-3, MB1-3, PMB1-3, FG1-3, MG1-3, PMG1-3, FL1-3, ML1-3, PML1-3, FM1-3, MM1-3, and PMM1-3. Total RNA was extracted using the Trizol reagent kit (Invitrogen, United States) according to the manufacturer’s protocol.

### Library Construction and Sequencing

The RNA quality was assessed on an Agilent 2,100 Bioanalyzer (Agilent Technologies, United States) and by agarose gel electrophoresis, then the total RNAs with RIN > 7.0 were used for library construction. Briefly, mRNA and ncRNA were retained by removing rRNAs using Ribo-ZeroTM Magnetic Kit (Epicentre, United States). The cDNA fragments were reverse-transcribed using DNA polymerase I and purified with a QiaQuick PCR extraction kit (Qiagen, Venlo, Netherlands). After end repair, poly(A) was added and ligated onto Illumina sequencing adapters. The cDNA libraries were sequenced on Illumina HiSeqTM 4,000 (Guangzhou Genedenovo Biotechnology Co. Ltd., China).

### Read Processing and Analysis of Differentially Expressed Genes

Clean reads were obtained by removing low-quality bases using fastp 0.18.0 software, while rRNA was removed using Bowtie 2.2.8. The remaining paired-end cleaned reads were mapped to the *C. semilaevis* reference genome (Cse_v1.0) using HISAT2 2.1.0 for transcript reconstruction and novel transcripts identification. FPKM (fragment per kilobase of transcript per million mapped reads) values were calculated using StringTie 1.3.1 to quantify expression abundance for the transcription region.

The differentially expressed genes (DEGs) were identified using DESeq2 software. All DEGs were mapped subjected to Gene Ontology (GO) analysis on the GO database (http://www.geneontology.org/) and KEGG database (http://www.kegg.jp), respectively, to infer functions of the DEGs. Gene numbers were calculated for every term and significantly enriched GO terms/KEGG pathways in DEGs compared to the genome background, as defined by hypergeometric tests. The GO terms or KEGG pathways with corrected FDR < 0.05 were considered significantly enriched.

### Validation of DEGs by qRT-PCR

Twelve DEGs (Supplementary Table S4) were selected for the qRT-PCR validation as previously described (Wang et al., 2018) using the *C. semilaevis* β-actin as the internal reference. Total RNA was reverse-transcribed into cDNA using the PrimeScript RT reagent Kit with gDNA Eraser (Takara Bio, Japan). The qPCR reaction was conducted using SYBR Premix Ex Taq (Takara Bio, Japan) in a 20 μL reaction on the ABI 7500 Fast Real-time PCR System (Applied Biosystems, United States). Fold change values for relative expression levels of the 12 genes were calculated using the 2^−ΔΔCt^ method (Livak and Schmittgen, 2001). The log2FC values retrieved by qPCR and RNA-seq counts were used for graphical presentation by GraphPad Prism 8.

### The Weighted Gene Co-expression Network Analysis for DEGs

Co-expression networks were constructed using WGCNA (v1.47) to describe the correlation patterns among DEGs across multiple samples and find modules of highly correlated to female-biased SSD in the R package following a previous procedure (Langfelder and Horvath, 2008). Briefly, DEGs were submitted for co-expression modules construction with the power = 8 and minModuleSize = 50. GO and KEGG pathway enrichment analysis were conducted for genes in each module to analyze the biological functions. Subsequently, module eigengenes were analyzed to determine the correlation relationship between modules and the growth performance of samples. Modules with the highest/lowest correlation values and *p* < 0.05 were considered positive-/negative-related, while the genes manifesting the closest relationships with other genes were recognized as hub genes. The network for hub genes characterization was generated by Gephi 0.9.2 (Bastian et al., 2009).

### Genomic DNA Library Construction, Sequencing, and Data Filtering

For interpreting the possible epigenetic mechanism involved in sexual size dimorphism, gonad and muscle tissues with more DEGs between different sexual individuals were submitted for whole-genome DNA methylation sequence analysis. The integrity of the extracted DNA was determined using NanoPhotometer^®^, spectrophotometer (IMPLEN, United States), and agarose gel electrophoresis. Thereafter, the DNA was fragmented into 100–300 bp by Sonication (Covaris, United States) and purified using the MiniElute PCR Purification Kit (QIAGEN, United States). The purified DNA fragments were ligated to methylate sequencing adapters after end repair with the “A” nucleotide. Subsequently, bisulfite conversion was conducted using a Methylation-Gold kit (ZYMO, United States) to convert unmethylated cytosine to uracil. Finally, the converted DNA fragments were PCR amplified and sequenced using Illumina HiSeqTM 2,500 at Gene Denovo Biotechnology Co. (Guangzhou, China).

### Methylation Level Analysis

Raw reads were filtered by removing reads containing >10% unknown nucleotides and low-quality reads containing >40% low-quality (Q-value ≤ 20) bases. The cleaned, filtered reads were mapped to the *C. semilaevis* reference genome using BSMAP 2.90. The methylated cytosines were called using a Perl script and tested using a previous algorithm (Lister et al., 2009). The methylation level was calculated based on the percentage of methylated cytosine in the whole genome, on each chromosome, and at different genomic regions for each sequence context (CG, CHG, and CHH). The methylation profiles at flanking 2 kb regions and gene body were plotted based on the average methylation levels for each window to assess distinct methylation patterns in different genomic regions.

### The Identification of Differentially Methylated Regions and Functional Enrichment Analysis for Differentially Methylated Genes

DMRs between two samples were identified using methylkit V1.4.1 with calling window size of 200 bp and the minimum read coverage of 4 bp. DMRs for each sequence context (CG, CHG, and CHH) based on this criteria: 1) For CG and CHG, numbers in each window ≥5, absolute values of the difference in methylation ratio ≥ 0.25, and q ≤ 0.05; 2) For CHH, numbers in a window ≥15, absolute values of the difference in methylation ratio ≥0.15, and q ≤ 0.05; 3) For all C, numbers in a window ≥20, absolute values of the difference in methylation ratio ≥0.2, and q ≤ 0.05. Subsequently, GO and KEGG pathway enrichment analysis were conducted for DMGs.

### The Correlation of DNA Methylation and Gene Expression in Samples

Genes were categorized into four classes based on their expression levels to determine whether gene expression influences DNA methylation. These classes included non-expressed (RPKM ≤ 1), low-expressed (1 < RPKM ≤ 10), middle-expressed (10 < RPKM ≤ 100), and high-expressed groups (RPKM > 100).

Spearman’s correlation analysis was performed to discern the statistical relationships between DNA methylation and gene expression within ± 2 kb flanking regions and the gene body. A positive correlation was indicated by rho >0, while rho <0 indicated a negative correlation.

### Correlation of DMGs and DEGs Between Groups

Common genes between DMGs and DEGs were extracted, and their GO/KEGG enrichment analysis was conducted to explore the potential functions of DNA methylation responsible for DEGs.

### The Nine Quadrant Diagram for Module Genes by Combining Their Transcripts and DNA Methylation Levels

The genes from the positive or negative growth correlated modules were separately selected for the integration by combining their transcripts and DNA methylation levels within the R software. The value for DEG-selection was set at |log2FC| > 1, and the value for DMG-selection was set at |meth.diff| > 25.

## Data Availability

The whole transcriptome and methylome data presented in the study are deposited in the NCBI SRA repository, accession number PRJNA743138 and PRJNA741107, respectively.
