# Supplementary material for: Integration of Transcriptome and Methylome Highlights the Roles of Cell Cycle and Hippo Signaling Pathway in Flatfish Sexual Size Dimorphism
Source: Front Cell Dev Biol. 2021 Dec 2;9:743722. doi: 10.3389/fcell.2021.743722 (PMC8675331; doi:10.3389/fcell.2021.743722)
Supplement: Supplementary file 1 [file DataSheet1.pdf]

Supplementary files for

Integration of transcriptome and methylome highlights the roles of cell cycle and hippo signaling pathway in fish sexual size dimorphism

Na Wang[a, b, c*], Qian Yang[a, d], Jialin Wang[a, d], Rui Shi[a, d], Ming Li[a, b], Jin Gao[d], Wenteng Xu[a, b], Yingming Yang[a, b, c], Yadong Chen[a, b], Songlin Chen[a, b*]

[a] Key Laboratory for Sustainable Development of Marine Fisheries, Ministry of Agriculture and Rural Affairs, Yellow Sea Fisheries Research Institute, Chinese Academy of Fishery Sciences, Qingdao 266071, China

[b] Laboratory for Marine Fisheries Science and Food Production Processes, Qingdao National Laboratory for Marine Science and Technology, Qingdao 266235, China

[c] Shandong Key Laboratory of Marine Fisheries Biotechnology and Genetic Breeding, Qingdao 266071, China

[d] College of Fisheries and Life Science, Shanghai Ocean University, Shanghai 201306, China

[e] Hainan Academy of Ocean and Fisheries Sciences, Haikou 570203, China

*Correspondence:

Profs. Na Wang and Songlin Chen
Yellow Sea Fisheries Research Institute
Chinese Academy of Fishery Sciences
106 Nanjing Road
Qingdao 266071, China
Tel: (+)86-532-85831605;
Fax: (+)86-532-85811514;
Email: wangna@ysfri.ac.cn; chensl@ysfri.ac.cn

Figure S1. The reads filter statistics in the whole transcriptomic analysis.

Figure S2. The PCA analysis for the whole transcriptome.

Figure S3. The top 20 GO terms in the brain.

Figure S4. The top 20 GO terms in the liver.

Figure S5. The top 20 GO terms in the gonad.

Figure S6. The top 20 GO terms in the muscle.

Figure S7. The genes' expression pattern in the turquoise and brown modules and their relationship between module membership and gene significance for body weight.

Figure S8. The GO terms enrichment in the turquoise module.

Figure S9. The GO terms enrichment in the brown module.

Figure S10. The sequencing depth and coverage analysis in WGBS.

Figure S11. The expression pattern of important epigenetic regulatory factors.

Figure S12. The demonstration of DMRs in the methylome and their functional KEGG enrichment analysis.

Figure S13. The KEGG enrichment analysis for the DMGs.

Figure S14. The Spearman's correlation between DNA methylation and gene expression within the samples.

Figure S15. The overlapped genes in DMGs and DEGs, and their trends calculation.

Table S1. The reads information in the whole transcriptomic analysis.

Table S2. The KEGG enrichment in four tissues.

Table S3. The sample growth trait used in WGCNA.

Table S4. The primers used in present study.

Table S5. The sequencing data information in the WGBS.

Table S6. The genomic DNA methylation levels in 18 samples.

Figure S1.The reads filter statistics in the whole transcriptomic analysis.

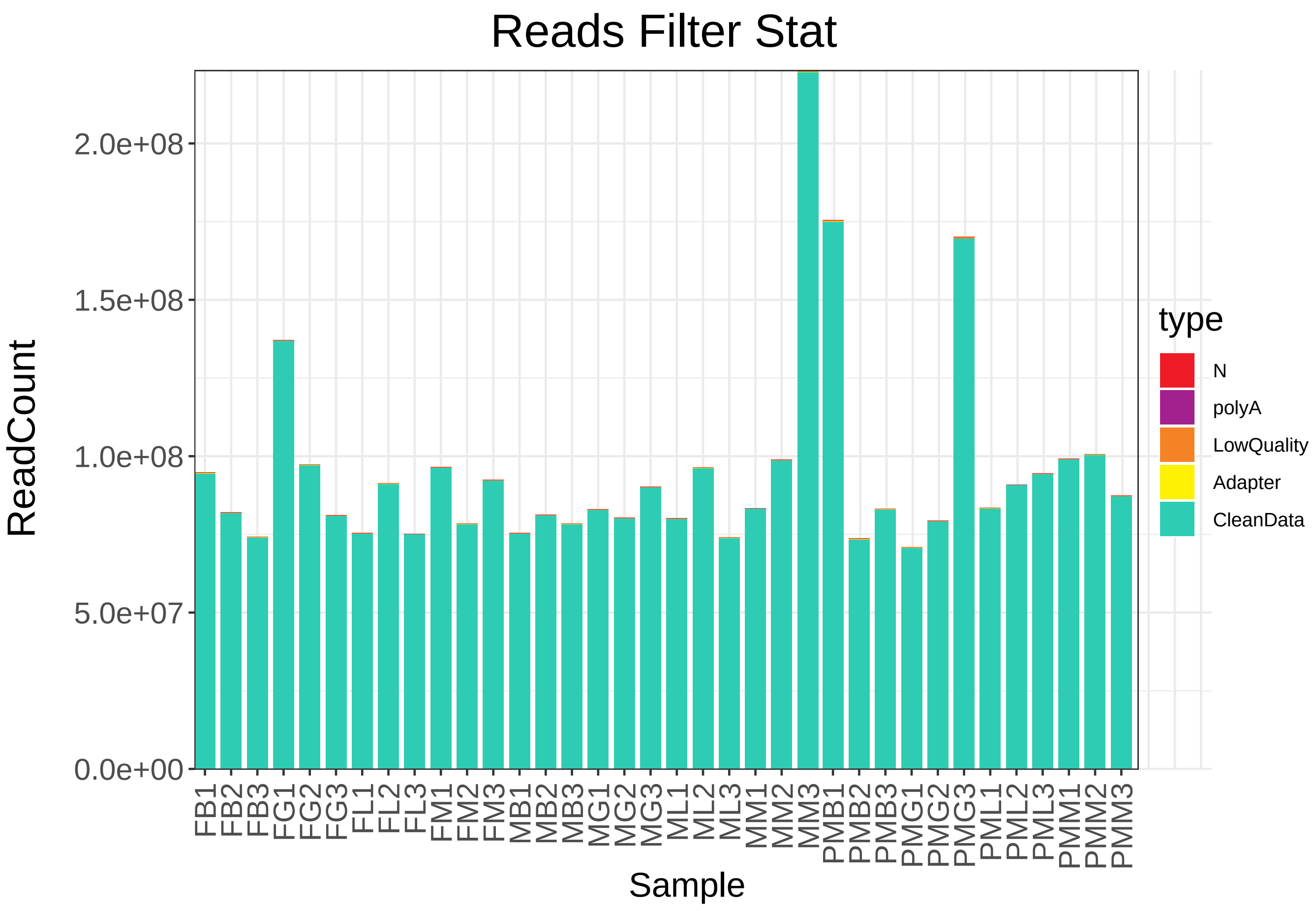

Figure S2. The PCA analysis for the whole transcriptome.

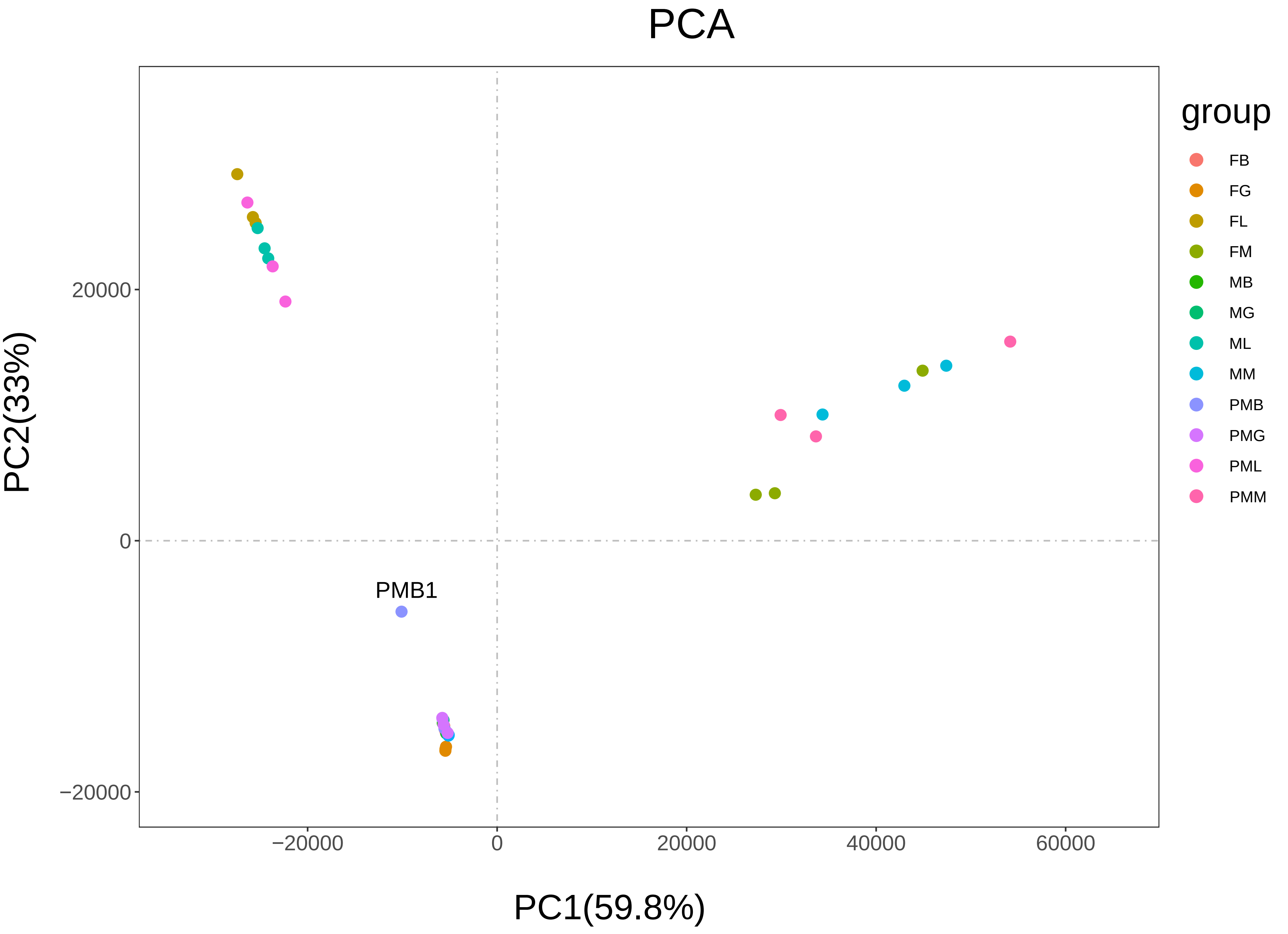

Figure S3. The top 20 GO terms in the brain.

A

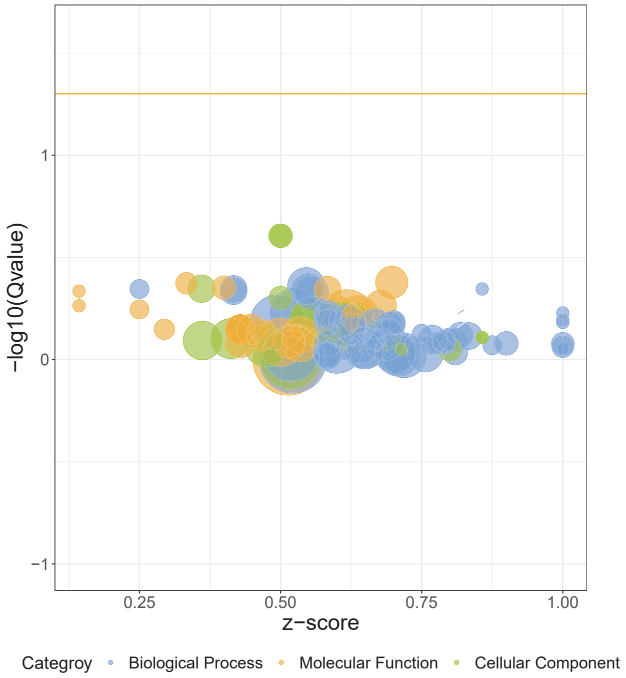

Top 20 GO terms in MB-vs-FB

ID	Descrption
GO:0051179	localization
GO:0044765	single−organism transport
GO:0006811	ion transport
GO:0033036	macromolecule localization
GO:0006810	transport
GO:1902578	single−organism localization
GO:0051234	establishment of localization
GO:0030154	cell differentiation
GO:0043228	non−membrane−bounded organelle
GO:0043232	intracellular non−membrane−bounded organelle
GO:0044422	organelle part
GO:0044446	intracellular organelle part
GO:0005515	protein binding
GO:0022892	substrate−specific transporter activity
GO:0005215	transporter activity
GO:0046914	transition metal ion binding
GO:0015075	ion transmembrane transporter activity
GO:0003676	nucleic acid binding
GO:0022891	substrate−specific transmembrane transporter activity
GO:0022857	transmembrane transporter activity

B

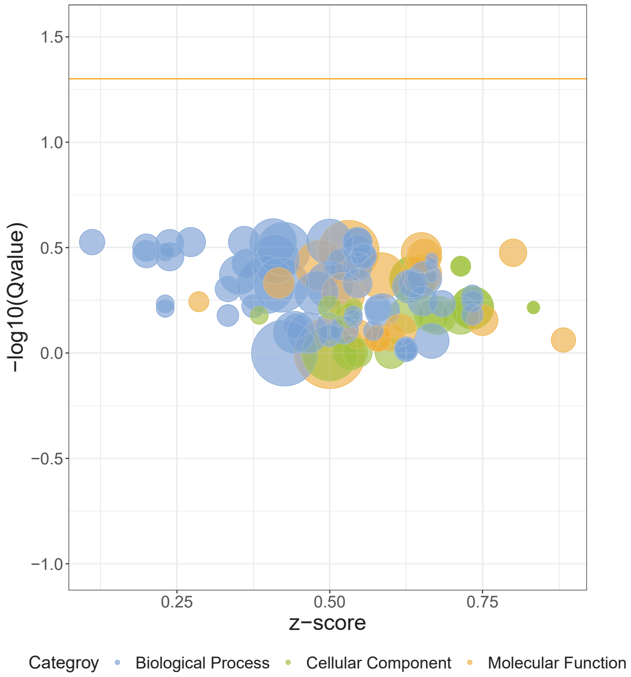

Top 20 GO terms in MB-vs-PMB

ID	Descrption
GO:0044237	cellular metabolic process
GO:0044238	primary metabolic process
GO:0006807	nitrogen compound metabolic process
GO:0006464	cellular protein modification process
GO:1901360	organic cyclic compound metabolic process
GO:0036211	protein modification process
GO:0090304	nucleic acid metabolic process
GO:0006725	cellular aromatic compound metabolic process
GO:0006139	nucleobase−containing compound metabolic process
GO:0008152	metabolic process
GO:0043412	macromolecule modification
GO:0016070	RNA metabolic process
GO:0046483	heterocycle metabolic process
GO:0006796	phosphate−containing compound metabolic process
GO:0034641	cellular nitrogen compound metabolic process
GO:0006793	phosphorus metabolic process
GO:0005488	binding
GO:0043169	cation binding
GO:0046914	transition metal ion binding
GO:0016787	hydrolase activity

C

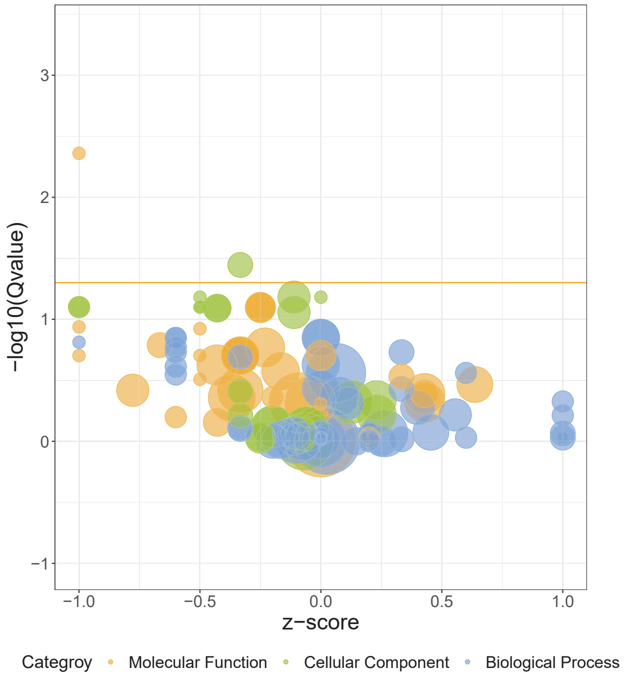

Top 20 GO terms in PMB-vs-FB

ID	Descrption
GO:0044765	single−organism transport
GO:1902578	single−organism localization
GO:0006461	protein complex assembly
GO:0005856	cytoskeleton
GO:0044446	intracellular organelle part
GO:0015630	microtubule cytoskeleton
GO:0044430	cytoskeletal part
GO:0031988	membrane−bounded vesicle
GO:0031982	vesicle
GO:0044428	nuclear part
GO:0005634	nucleus
GO:0043228	non−membrane−bounded organelle
GO:0043232	intracellular non−membrane−bounded organelle
GO:0044422	organelle part
GO:0044389	ubiquitin−like protein ligase binding
GO:0016462	pyrophosphatase activity
GO:0016818	hydrolase activity, acting on acid anhydrides, in phosphorus−containing anhydrides
GO:0016817	hydrolase activity, acting on acid anhydrides
GO:0019899	enzyme binding
GO:0005198	structural molecule activity

Figure S4. The top 20 GO terms in the liver.

A

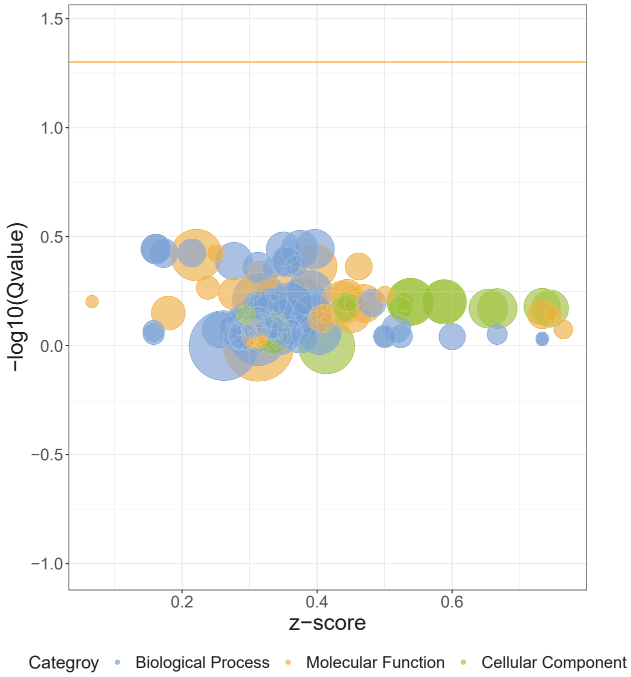

Top 20 GO terms in ML-vs-FL

ID	Descrption
GO:0051179	localization
GO:0051234	establishment of localization
GO:0006810	transport
GO:0006796	phosphate−containing compound metabolic process
GO:0006793	phosphorus metabolic process
GO:1902578	single−organism localization
GO:0044765	single−organism transport
GO:0019538	protein metabolic process
GO:0006464	cellular protein modification process
GO:0036211	protein modification process
GO:0031323	regulation of cellular metabolic process
GO:0043412	macromolecule modification
GO:0080090	regulation of primary metabolic process
GO:0044238	primary metabolic process
GO:0016788	hydrolase activity, acting on ester bonds
GO:0003824	catalytic activity
GO:0097159	organic cyclic compound binding
GO:0046914	transition metal ion binding
GO:0016787	hydrolase activity
GO:0016772	transferase activity, transferring phosphorus−containing groups

B

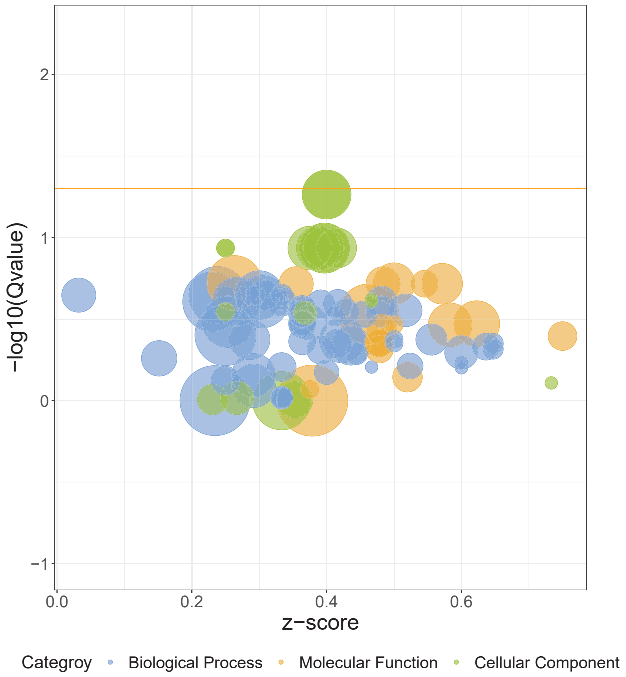

Top 20 GO terms in ML-vs-PML

ID	Descrption
GO:0008152	metabolic process
GO:0044238	primary metabolic process
GO:0044710	single−organism metabolic process
GO:0006796	phosphate−containing compound metabolic process
GO:0044424	intracellular part
GO:0005622	intracellular
GO:0005623	cell
GO:0044464	cell part
GO:0043226	organelle
GO:0043229	intracellular organelle
GO:0043227	membrane−bounded organelle
GO:0043231	intracellular membrane−bounded organelle
GO:0043228	non−membrane−bounded organelle
GO:0043232	intracellular non−membrane−bounded organelle
GO:0003824	catalytic activity
GO:0043167	ion binding
GO:0043169	cation binding
GO:0046914	transition metal ion binding
GO:0016787	hydrolase activity
GO:0046872	metal ion binding

C

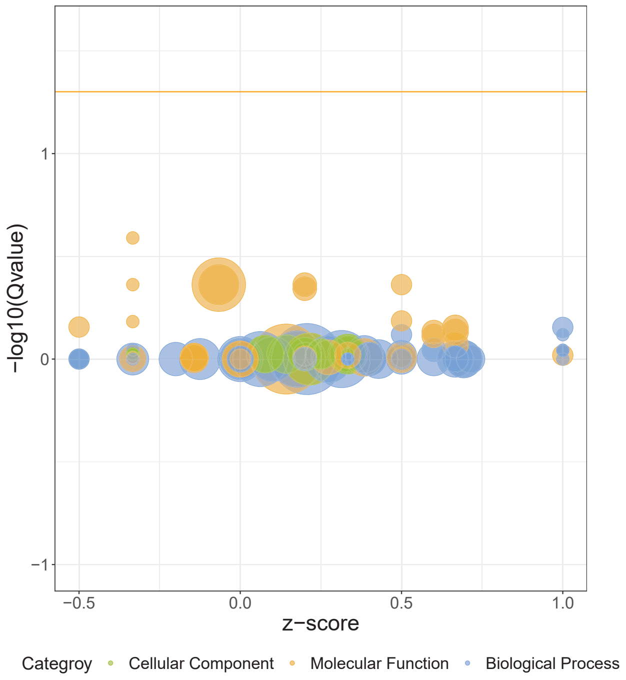

Top 20 GO terms in PML-vs-FL

ID	Descrption
GO:0070887	cellular response to chemical stimulus
GO:0010033	response to organic substance
GO:0071310	cellular response to organic substance
GO:0042221	response to chemical
GO:0007049	cell cycle
GO:0016798	hydrolase activity, acting on glycosyl bonds
GO:0003824	catalytic activity
GO:0016787	hydrolase activity
GO:0070011	peptidase activity, acting on L−amino acid peptides
GO:0004175	endopeptidase activity
GO:0016791	phosphatase activity
GO:0008233	peptidase activity
GO:0016491	oxidoreductase activity
GO:0042578	phosphoric ester hydrolase activity
GO:0016788	hydrolase activity, acting on ester bonds
GO:0004872	receptor activity
GO:0005215	transporter activity
GO:0038023	signaling receptor activity
GO:0004871	signal transducer activity
GO:0060089	molecular transducer activity

Figure S5. The top 20 GO terms in the gonad.

A

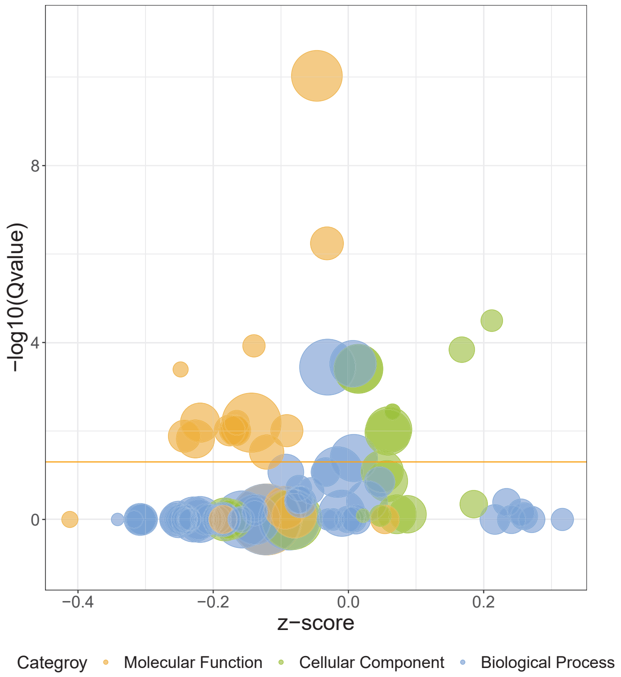

Top 20 GO terms in MG-vs-FG

ID	Descrption
GO:0044238	primary metabolic process
GO:0008152	metabolic process
GO:0044446	intracellular organelle part
GO:0044422	organelle part
GO:0005623	cell
GO:0044464	cell part
GO:0043228	non−membrane−bounded organelle
GO:0043232	intracellular non−membrane−bounded organelle
GO:0005622	intracellular
GO:0003824	catalytic activity
GO:0016740	transferase activity
GO:0016772	transferase activity, transferring phosphorus−containing groups
GO:0016301	kinase activity
GO:0005488	binding
GO:0043167	ion binding
GO:0046914	transition metal ion binding
GO:0001883	purine nucleoside binding
GO:0032549	ribonucleoside binding
GO:0032550	purine ribonucleoside binding
GO:0097367	carbohydrate derivative binding

B

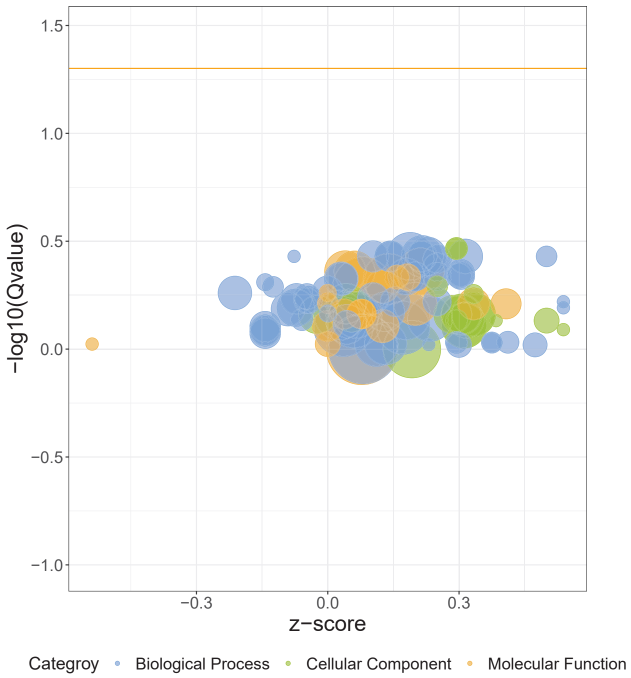

Top 20 GO terms in MG-vs-PMG

ID	Descrption
GO:0044238	primary metabolic process
GO:0051179	localization
GO:0051234	establishment of localization
GO:0006810	transport
GO:0043412	macromolecule modification
GO:0006464	cellular protein modification process
GO:1901360	organic cyclic compound metabolic process
GO:0036211	protein modification process
GO:0033036	macromolecule localization
GO:0043933	macromolecular complex subunit organization
GO:0044765	single−organism transport
GO:1902578	single−organism localization
GO:0006796	phosphate−containing compound metabolic process
GO:0090304	nucleic acid metabolic process
GO:0006793	phosphorus metabolic process
GO:0043228	non−membrane−bounded organelle
GO:0043232	intracellular non−membrane−bounded organelle
GO:0097159	organic cyclic compound binding
GO:0043167	ion binding
GO:0043169	cation binding

C

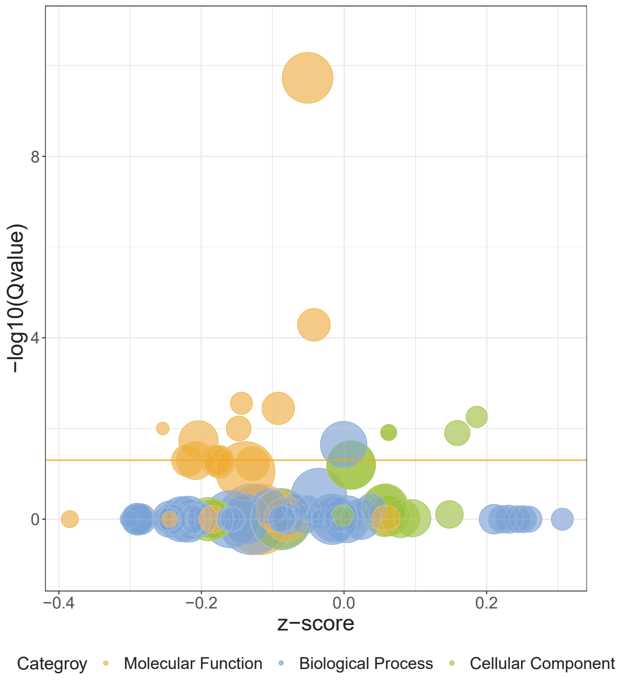

Top 20 GO terms in PMG-vs-FG

ID	Descrption
GO:0044238	primary metabolic process
GO:0044446	intracellular organelle part
GO:0043228	non−membrane−bounded organelle
GO:0043232	intracellular non−membrane−bounded organelle
GO:0044422	organelle part
GO:0003824	catalytic activity
GO:0016740	transferase activity
GO:0016772	transferase activity, transferring phosphorus−containing groups
GO:0016787	hydrolase activity
GO:0046914	transition metal ion binding
GO:0016301	kinase activity
GO:0043167	ion binding
GO:0043169	cation binding
GO:0046872	metal ion binding
GO:0097367	carbohydrate derivative binding
GO:0001883	purine nucleoside binding
GO:0032549	ribonucleoside binding
GO:0032550	purine ribonucleoside binding
GO:0001882	nucleoside binding
GO:0036094	small molecule binding

Figure S6. The top 20 GO terms in the muscle.

A

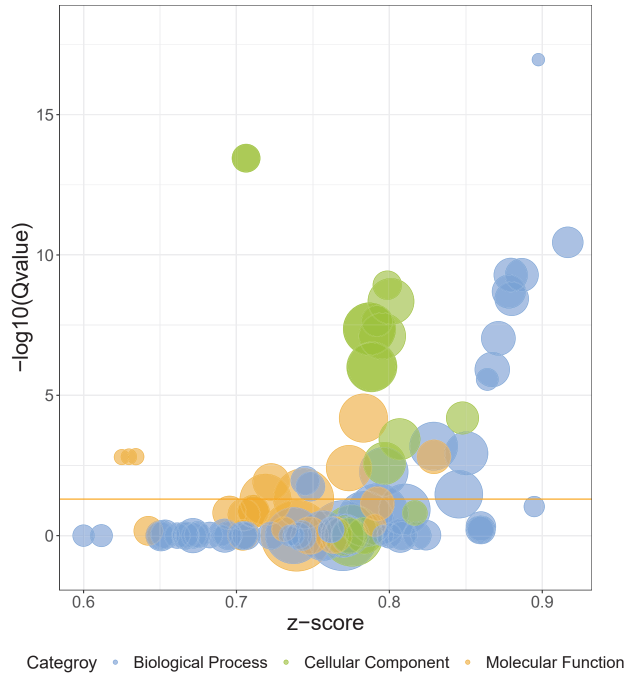

Top 20 GO terms in MM-vs-FM

ID	Descrption
GO:0006259	DNA metabolic process
GO:0090304	nucleic acid metabolic process
GO:0006725	cellular aromatic compound metabolic process
GO:0006139	nucleobase−containing compound metabolic process
GO:0046483	heterocycle metabolic process
GO:1901360	organic cyclic compound metabolic process
GO:0034641	cellular nitrogen compound metabolic process
GO:0006807	nitrogen compound metabolic process
GO:0006996	organelle organization
GO:0043228	non−membrane−bounded organelle
GO:0043232	intracellular non−membrane−bounded organelle
GO:0044446	intracellular organelle part
GO:0043229	intracellular organelle
GO:0044422	organelle part
GO:0005623	cell
GO:0044464	cell part
GO:0043226	organelle
GO:0005622	intracellular
GO:0044424	intracellular part
GO:0032991	macromolecular complex

B

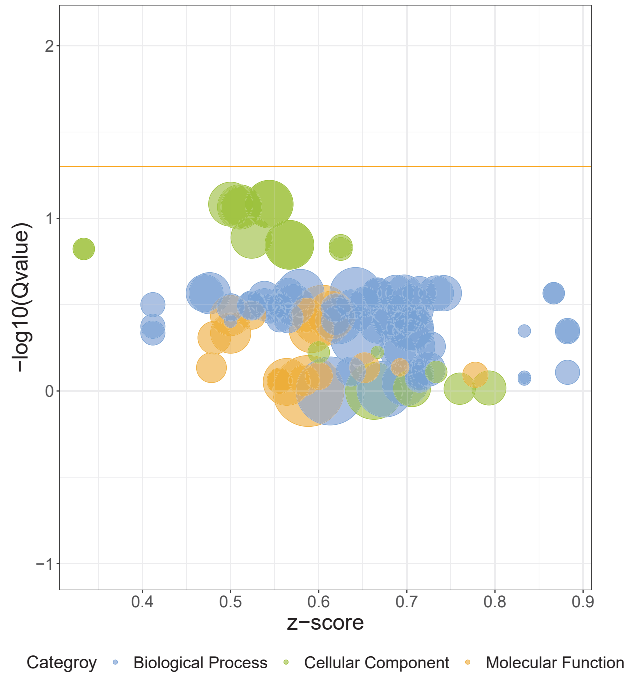

Top 20 GO terms in MM-vs-PMM

ID	Descrption
GO:0071704	organic substance metabolic process
GO:0043170	macromolecule metabolic process
GO:0051179	localization
GO:0051234	establishment of localization
GO:0032502	developmental process
GO:0044767	single−organism developmental process
GO:0032501	multicellular organismal process
GO:0044707	single−multicellular organism process
GO:0044424	intracellular part
GO:0005622	intracellular
GO:0043229	intracellular organelle
GO:0043226	organelle
GO:0043231	intracellular membrane−bounded organelle
GO:0043227	membrane−bounded organelle
GO:0005623	cell
GO:0044464	cell part
GO:0044444	cytoplasmic part
GO:0005737	cytoplasm
GO:0043228	non−membrane−bounded organelle
GO:0043232	intracellular non−membrane−bounded organelle

C

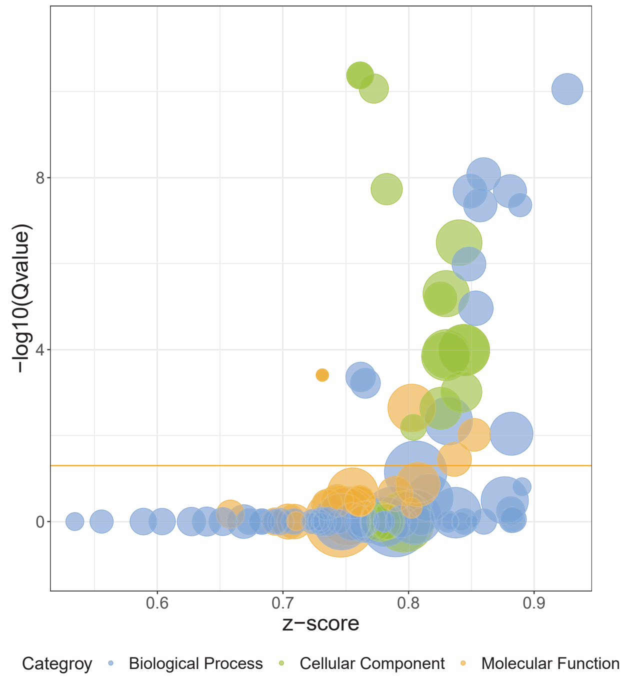

Top 20 GO terms in PMM-vs-FM

ID	Descrption
GO:0090304	nucleic acid metabolic process
GO:0006725	cellular aromatic compound metabolic process
GO:1901360	organic cyclic compound metabolic process
GO:0006139	nucleobase−containing compound metabolic process
GO:0046483	heterocycle metabolic process
GO:0006996	organelle organization
GO:0034641	cellular nitrogen compound metabolic process
GO:0006807	nitrogen compound metabolic process
GO:0043228	non−membrane−bounded organelle
GO:0043232	intracellular non−membrane−bounded organelle
GO:0044446	intracellular organelle part
GO:0044422	organelle part
GO:0043229	intracellular organelle
GO:0043226	organelle
GO:0032991	macromolecular complex
GO:0005623	cell
GO:0044464	cell part
GO:0005622	intracellular
GO:0044424	intracellular part
GO:0016462	pyrophosphatase activity

Figure S7. The genes' expression pattern in the turquoise and brown modules and their relationship between module membership and gene significance for body weight.

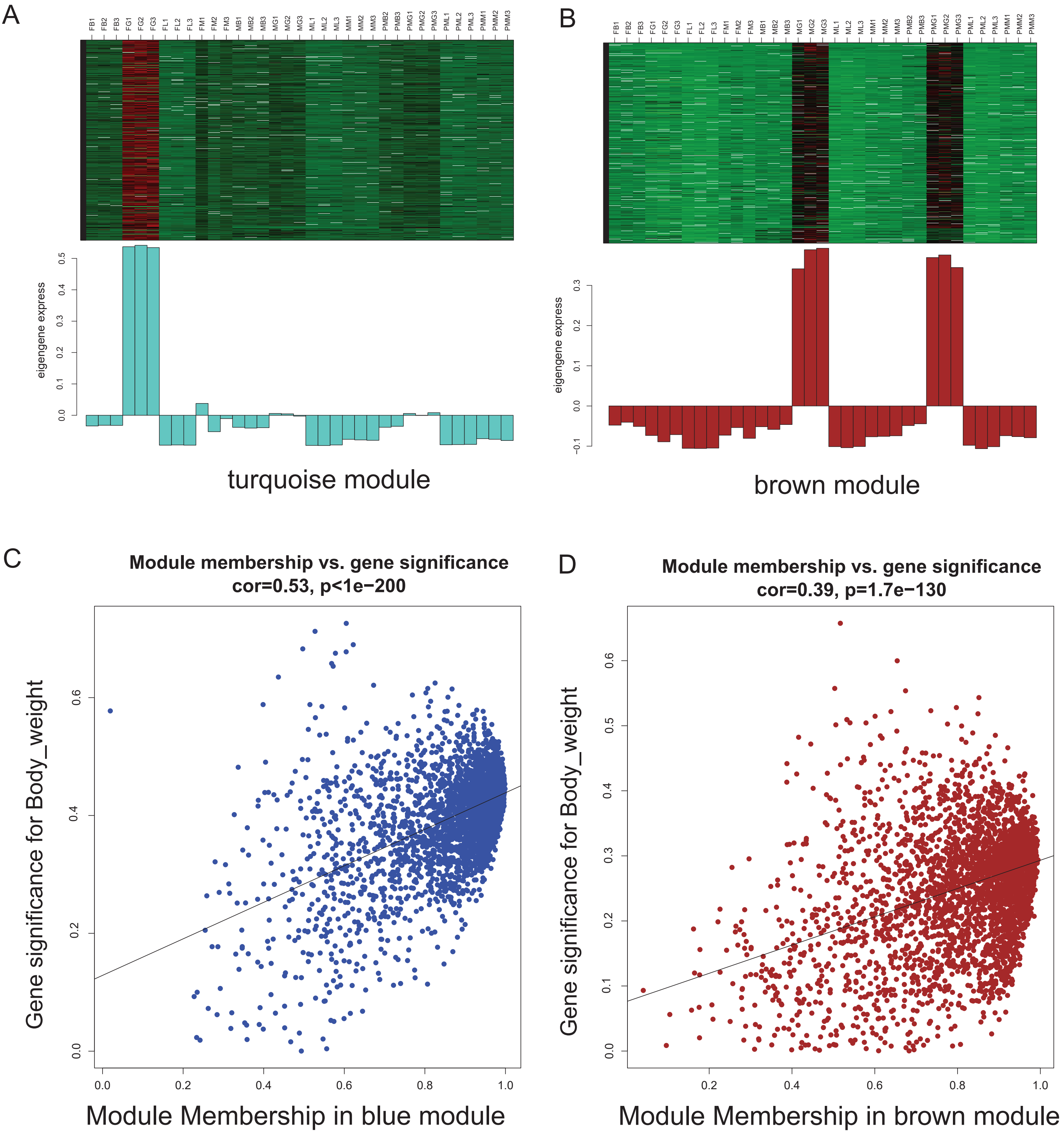

Figure S8. The GO terms enrichment in the turquoise module.

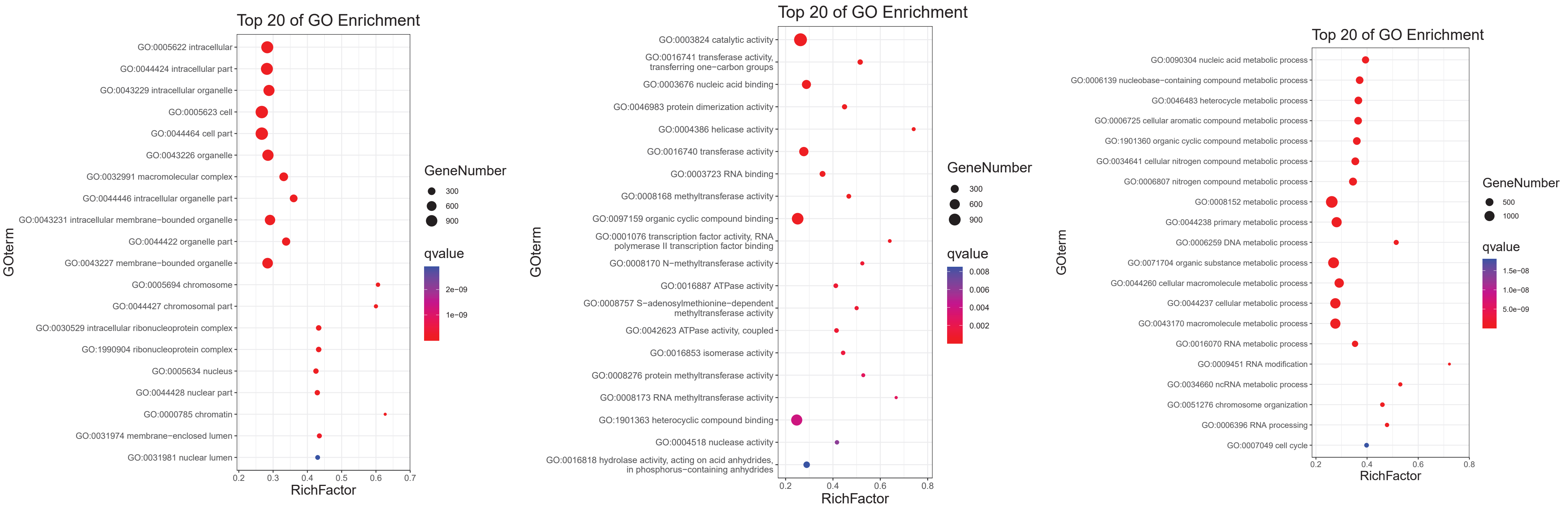

Figure S9. The GO terms enrichment in the brown module.

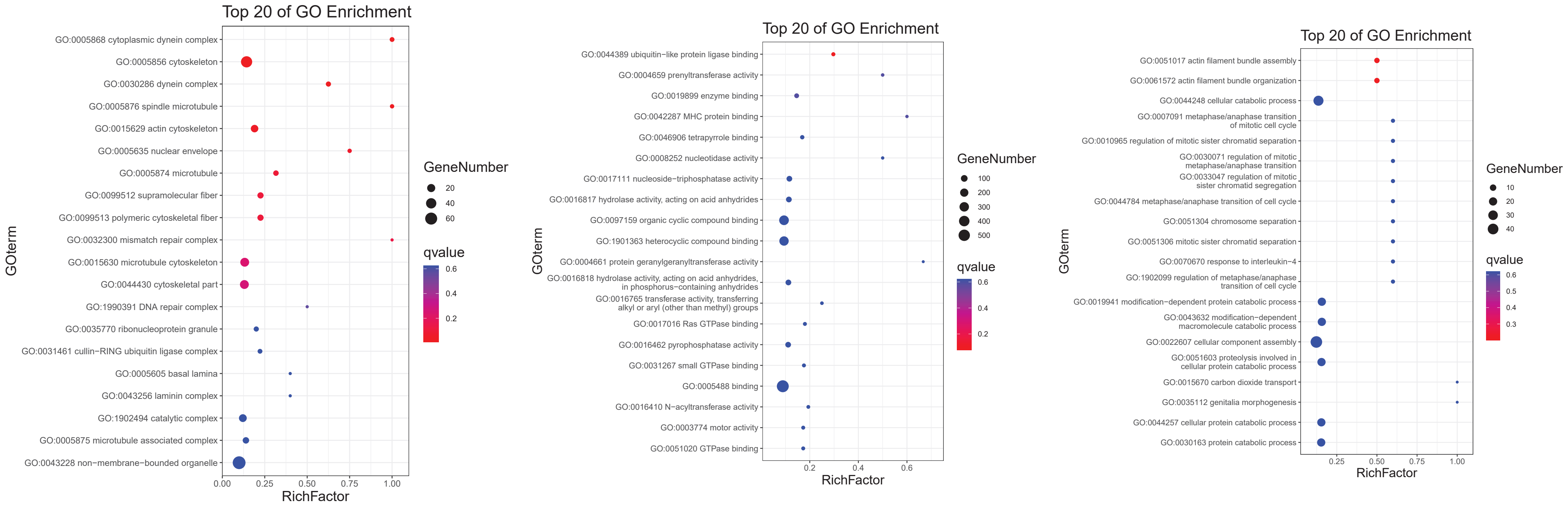

Figure S10. The sequencing depth and coverage analysis in WGBS.

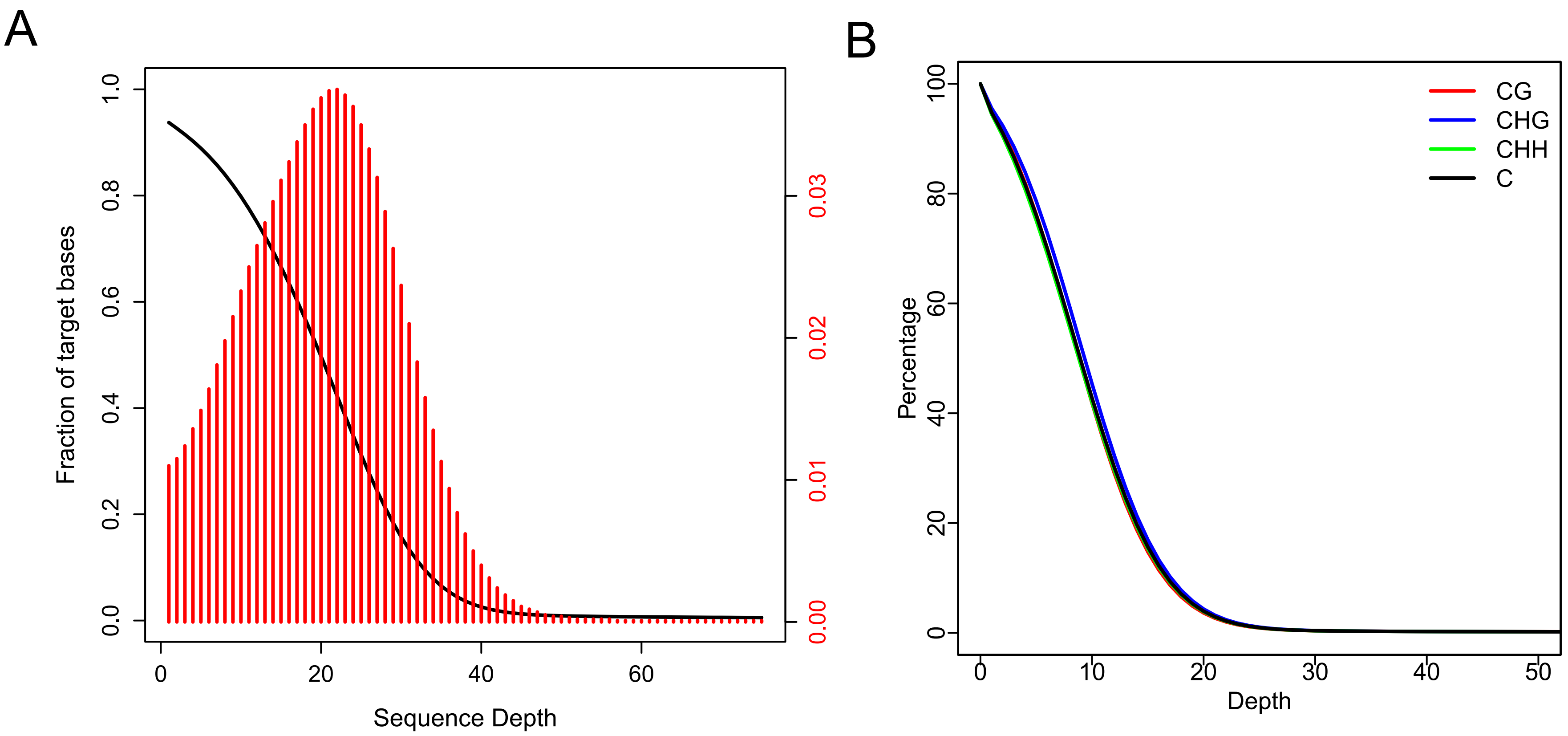

Figure S11. The expression pattern of important epigenetic regulatory factors.

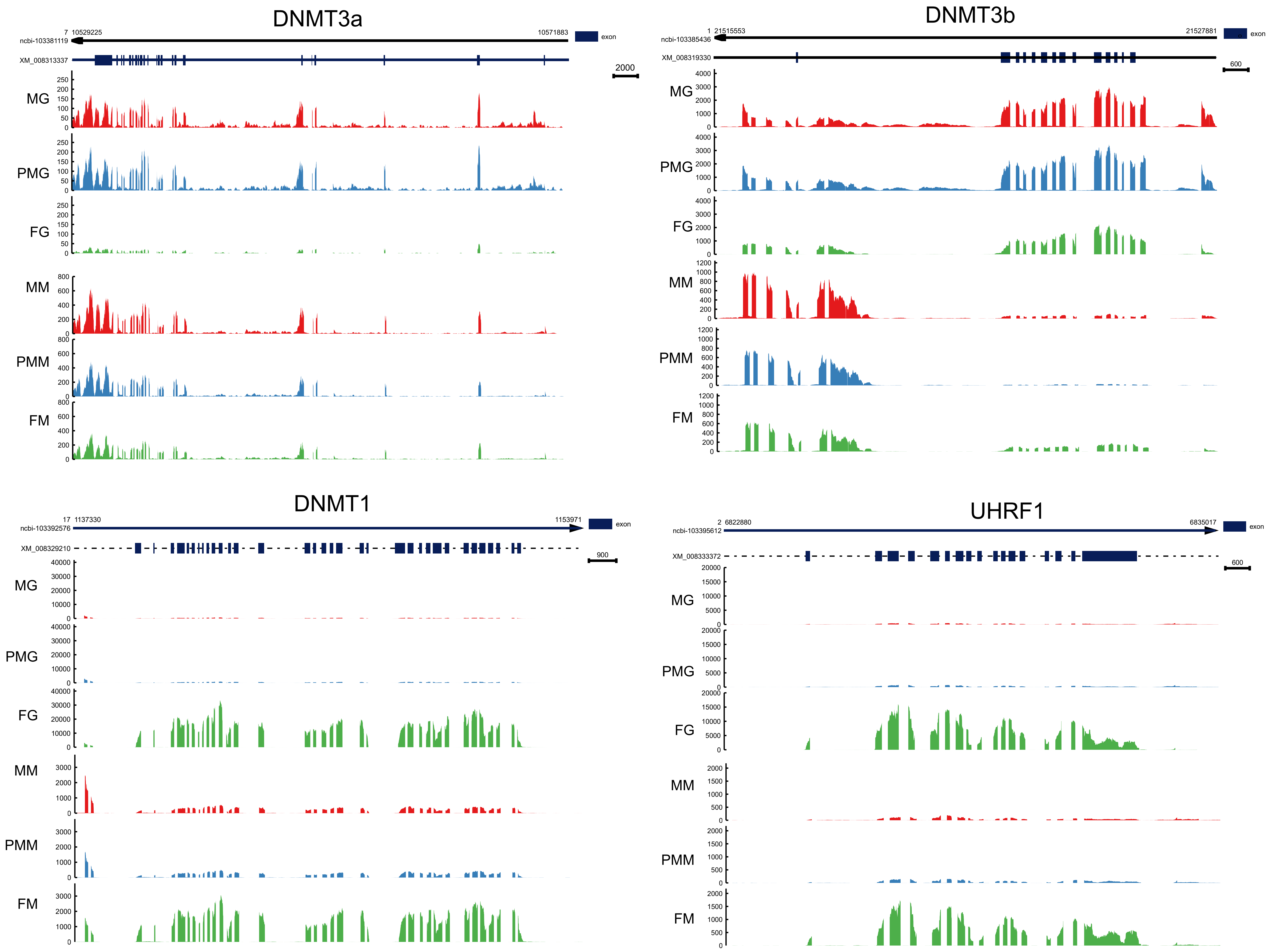

Figure S12. The demonstration of DMRs in the methylome and their functional KEGG enrichment analysis.

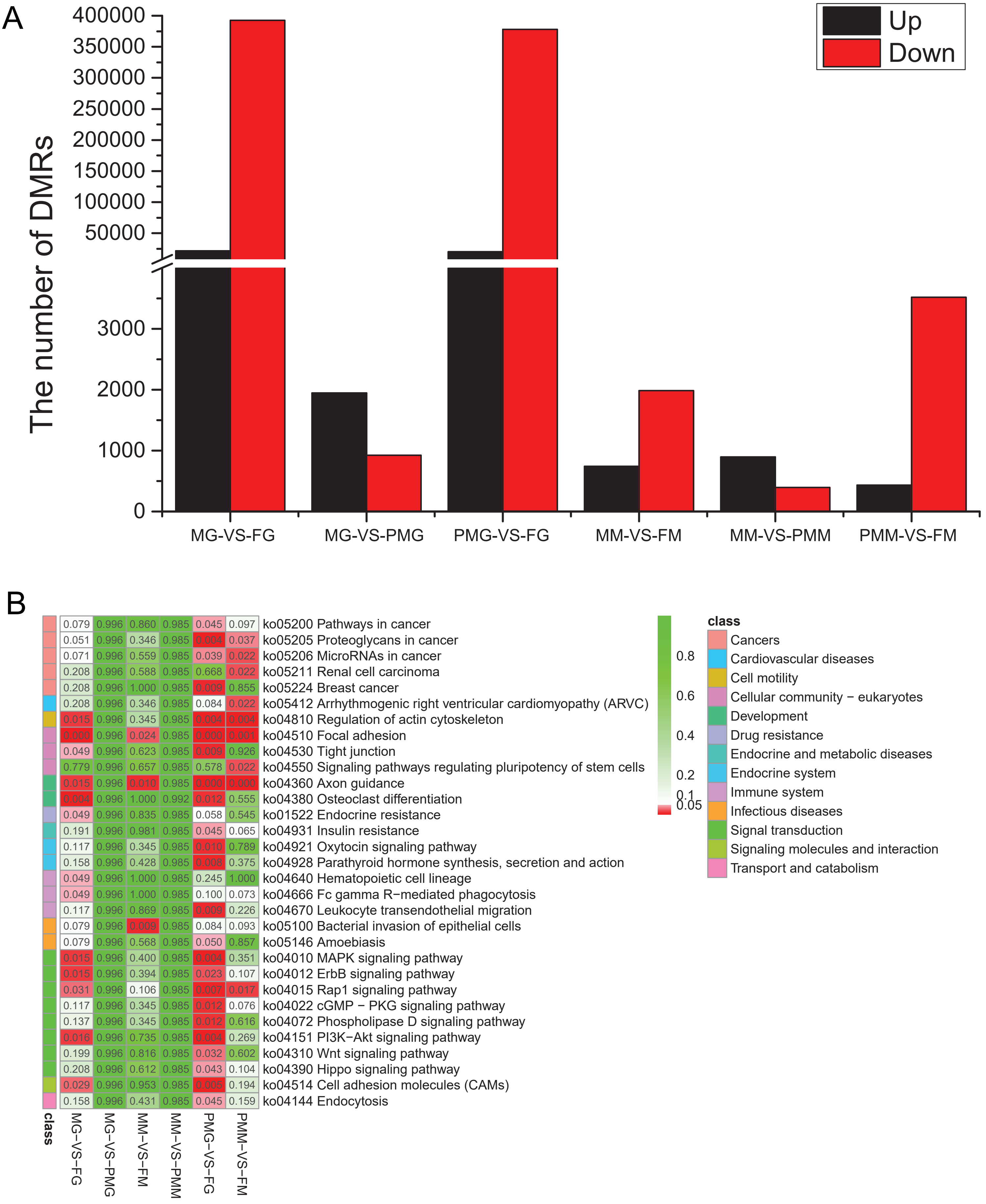

Figure S13. The KEGG enrichment analysis for the DMGs.

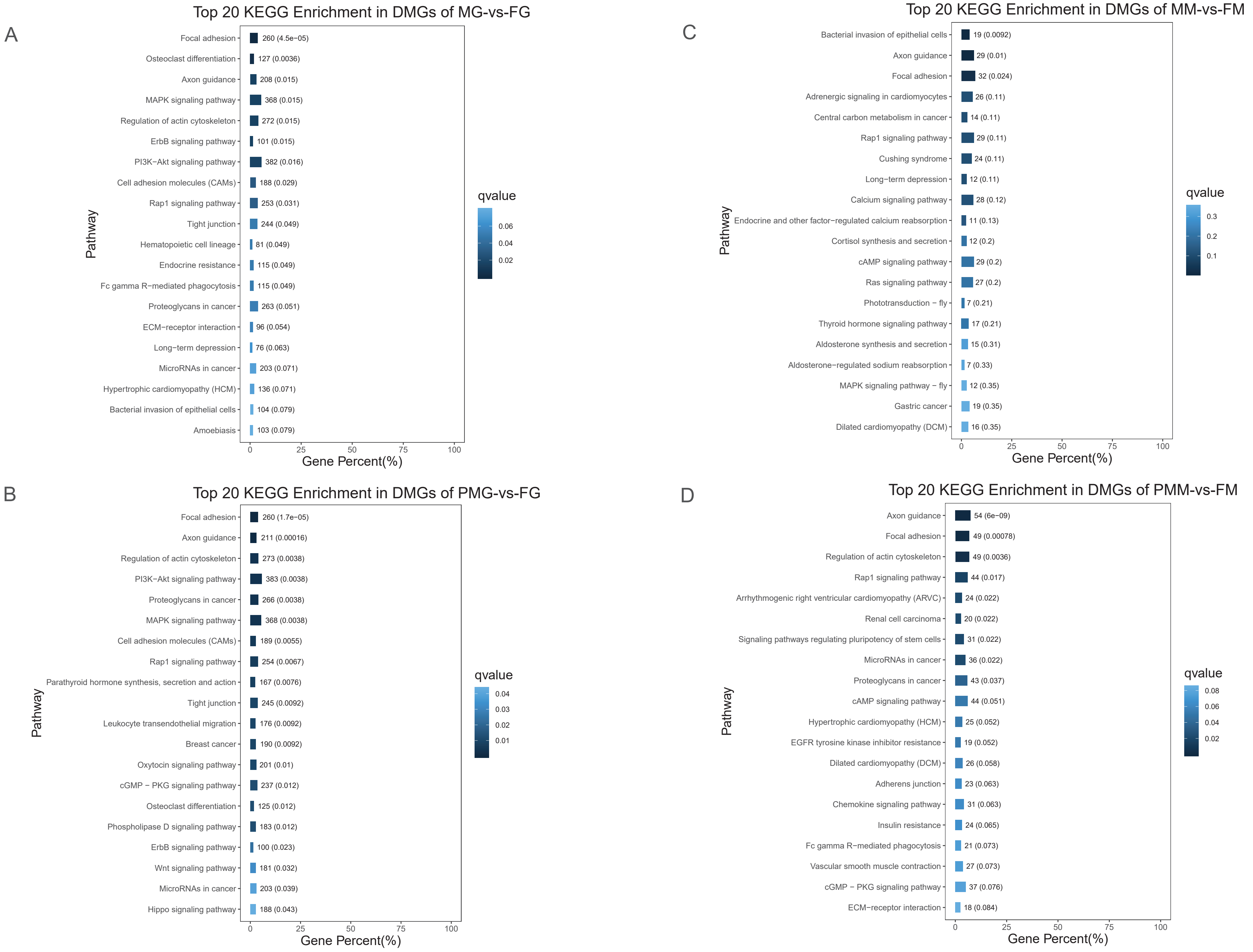

Figure S14. The Spearman's correlation between DNA methylation and gene expression within the samples.

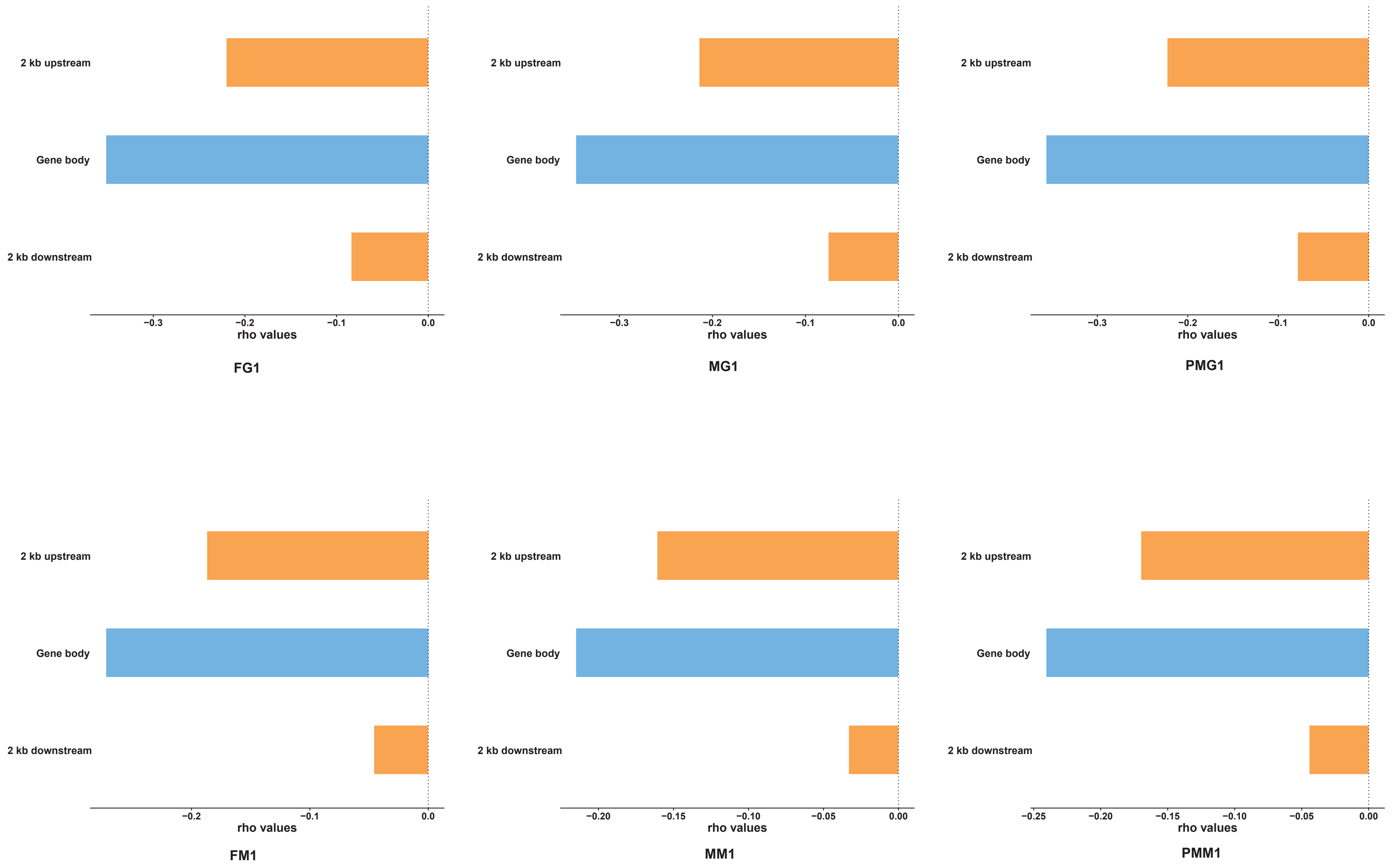

Figure S15. The overlapped genes in DMGs and DEGs, and their trends calculation.

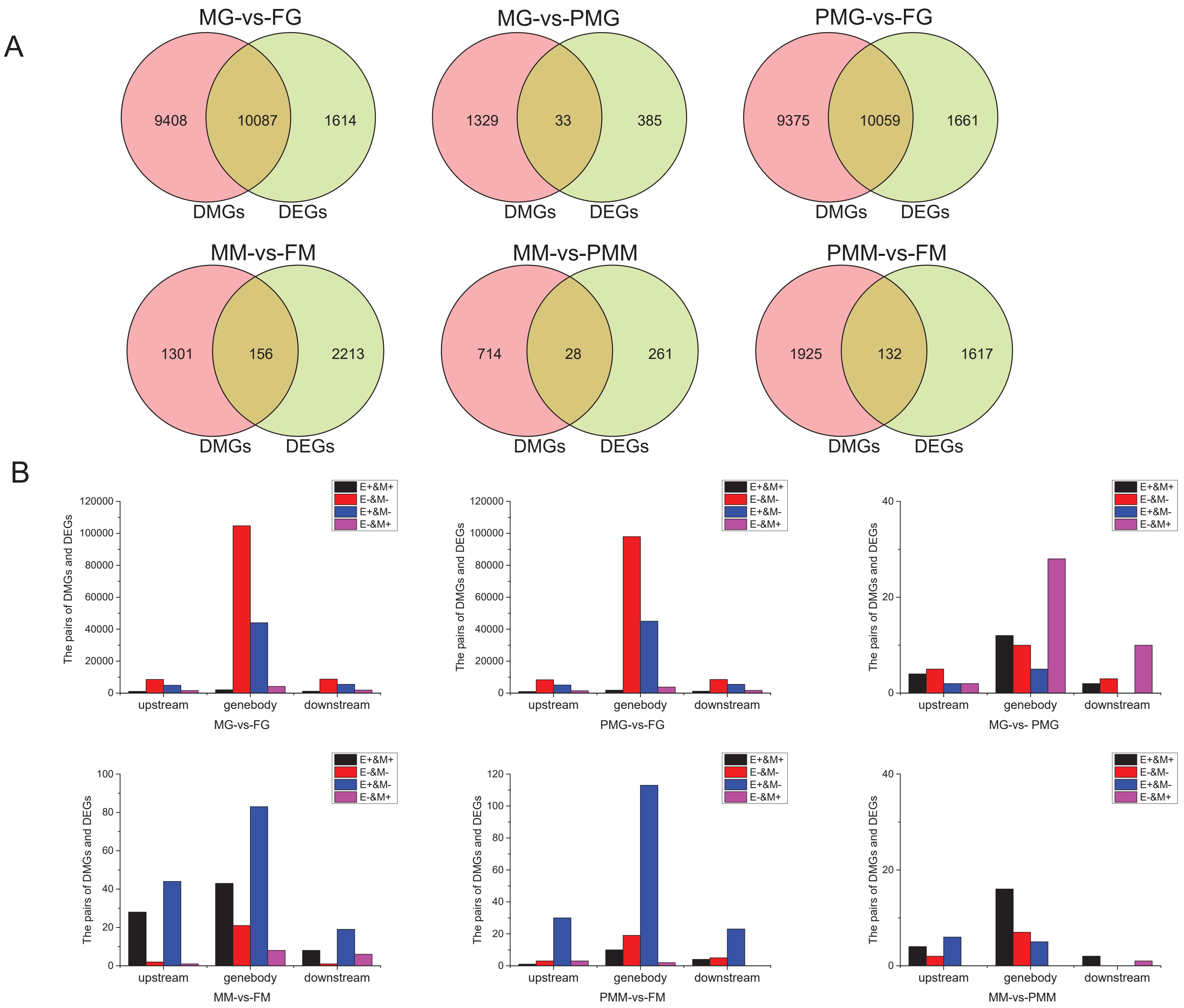

Table S1. The reads information in the whole transriptomi analysis.

Sample	RawDatas	CleanData(%)	Adapter(%)	LowQuality(%)	polyA(%)	N(%)	CleanData
FB1	94811290	94510580 (99.68%)	102736 (0.11%)	197792 (0.21%)	0 (0.00%)	182 (0.00%)	94510580
FB2	82084128	81845872 (99.71%)	77302 (0.09%)	160782 (0.20%)	0 (0.00%)	172 (0.00%)	81845872
FB3	74231542	74030202 (99.73%)	48412 (0.07%)	152928 (0.21%)	0 (0.00%)	0 (0.00%)	74030202
FG1	137107874	136876838 (99.83%)	58314 (0.04%)	172722 (0.13%)	0 (0.00%)	0 (0.00%)	136876838
FG2	97318682	97195282 (99.87%)	29112 (0.03%)	94272 (0.10%)	0 (0.00%)	16 (0.00%)	97195282
FG3	81040072	80898190 (99.82%)	26858 (0.03%)	115024 (0.14%)	0 (0.00%)	0 (0.00%)	80898190
FL1	75427550	75288994 (99.82%)	27904 (0.04%)	110652 (0.15%)	0 (0.00%)	0 (0.00%)	75288994
FL2	91339940	91160896 (99.80%)	33480 (0.04%)	145548 (0.16%)	0 (0.00%)	16 (0.00%)	91160896
FL3	75135958	75055356 (99.89%)	15632 (0.02%)	64806 (0.09%)	0 (0.00%)	164 (0.00%)	75055356
FM1	96499476	96316920 (99.81%)	46252 (0.05%)	136300 (0.14%)	0 (0.00%)	4 (0.00%)	96316920
FM2	78431672	78245342 (99.76%)	50234 (0.06%)	136096 (0.17%)	0 (0.00%)	0 (0.00%)	78245342
FM3	92414456	92190890 (99.76%)	68880 (0.07%)	154686 (0.17%)	0 (0.00%)	0 (0.00%)	92190890
MB1	75406168	75182548 (99.70%)	60234 (0.08%)	163386 (0.22%)	0 (0.00%)	0 (0.00%)	75182548
MB2	81316698	81051236 (99.67%)	73472 (0.09%)	191990 (0.24%)	0 (0.00%)	0 (0.00%)	81051236
MB3	78486654	78281698 (99.74%)	61030 (0.08%)	143766 (0.18%)	0 (0.00%)	160 (0.00%)	78281698
MG1	82980228	82778660 (99.76%)	51838 (0.06%)	149730 (0.18%)	0 (0.00%)	0 (0.00%)	82778660
MG2	80336438	80110742 (99.72%)	59842 (0.07%)	165854 (0.21%)	0 (0.00%)	0 (0.00%)	80110742
MG3	90271024	90055304 (99.76%)	48574 (0.05%)	167144 (0.19%)	0 (0.00%)	2 (0.00%)	90055304
ML1	80121430	79968532 (99.81%)	35824 (0.04%)	116838 (0.15%)	0 (0.00%)	236 (0.00%)	79968532
ML2	96400226	96250984 (99.85%)	32416 (0.03%)	116818 (0.12%)	0 (0.00%)	8 (0.00%)	96250984
ML3	74008444	73882874 (99.83%)	27832 (0.04%)	97738 (0.13%)	0 (0.00%)	0 (0.00%)	73882874
MM1	83387898	83242796 (99.83%)	35416 (0.04%)	109686 (0.13%)	0 (0.00%)	0 (0.00%)	83242796
MM2	98864352	98676840 (99.81%)	40722 (0.04%)	146790 (0.15%)	0 (0.00%)	0 (0.00%)	98676840
MM3	223195894	222800826 (99.82%)	99166 (0.04%)	295458 (0.13%)	0 (0.00%)	444 (0.00%)	222800826
PMB1	175465318	174938822 (99.70%)	137498 (0.08%)	388998 (0.22%)	0 (0.00%)	0 (0.00%)	174938822
PMB2	73684006	73444742 (99.68%)	66708 (0.09%)	172556 (0.23%)	0 (0.00%)	0 (0.00%)	73444742
PMB3	83210960	83003298 (99.75%)	61914 (0.07%)	145556 (0.17%)	0 (0.00%)	192 (0.00%)	83003298

PMG1	70916372	70739318 (99.75%)	32244 (0.05%)	144046 (0.20%)	0 (0.00%)	764 (0.00%)	70739318
PMG2	79364636	79156814 (99.74%)	48714 (0.06%)	159108 (0.20%)	0 (0.00%)	0 (0.00%)	79156814
PMG3	170060420	169579072 (99.72%)	111992 (0.07%)	369356 (0.22%)	0 (0.00%)	0 (0.00%)	169579072
PML1	83503244	83353394 (99.82%)	34556 (0.04%)	115102 (0.14%)	0 (0.00%)	192 (0.00%)	83353394
PML2	90939358	90838162 (99.89%)	17890 (0.02%)	83300 (0.09%)	0 (0.00%)	6 (0.00%)	90838162
PML3	94437886	94211768 (99.76%)	75426 (0.08%)	150682 (0.16%)	0 (0.00%)	10 (0.00%)	94211768
PMM1	99168972	98974518 (99.80%)	46942 (0.05%)	147266 (0.15%)	0 (0.00%)	246 (0.00%)	98974518
PMM2	100678354	100516610 (99.84%)	34612 (0.03%)	126942 (0.13%)	0 (0.00%)	190 (0.00%)	100516610
PMM3	87379998	87223556 (99.82%)	36346 (0.04%)	119886 (0.14%)	0 (0.00%)	210 (0.00%)	87223556

Table S2. The KEGG enrihment in four tissues.

Groups	ID	Descrption	Pvalue	Qvalue	Up	Down	fg_num	bg_num
MG-vs-FG	ko04110	Cell cycle	2.90E-05	0.009964	74	30	104	145
MG-vs-FG	ko05322	Systemic lupus erythematosus	0.00044	0.0548	31	19	50	66
MG-vs-FG	ko04610	Complement and coagulation cascades	0.00048	0.0548	5	37	42	54
MG-vs-FG	ko00260	Glycine, serine and threonine metabolism	0.00142	0.122397	17	16	33	42
MG-vs-FG	ko00053	Ascorbate and aldarate metabolism	0.00379	0.209123	9	11	20	24
MG-vs-FG	ko00240	Pyrimidine metabolism	0.00439	0.209123	46	25	71	104
MG-vs-FG	ko03013	RNA transport	0.00457	0.209123	71	40	111	170
MG-vs-FG	ko05216	Thyroid cancer	0.00486	0.209123	17	20	37	50
MG-vs-FG	ko04218	Cellular senescence	0.00593	0.226781	64	78	142	223
MG-vs-FG	ko03030	DNA replication	0.00873	0.275885	24	2	26	34
MG-vs-FG	ko04115	p53 signaling pathway	0.00882	0.275885	38	22	60	88
MG-vs-FG	ko00010	Glycolysis / Gluconeogenesis	0.01288	0.32574	22	30	52	76
MG-vs-FG	ko05150	Staphylococcus aureus infection	0.01308	0.32574	3	29	32	44
MG-vs-FG	ko00340	Histidine metabolism	0.01326	0.32574	11	6	17	21
MG-vs-FG	ko03440	Homologous recombination	0.02065	0.439776	22	5	27	37
MG-vs-FG	ko03320	PPAR signaling pathway	0.02109	0.439776	21	32	53	79
MG-vs-FG	ko05206	MicroRNAs in cancer	0.02423	0.439776	61	73	134	216
MG-vs-FG	ko05222	Small cell lung cancer	0.02435	0.439776	38	37	75	116
MG-vs-FG	ko01100	Metabolic pathways	0.02445	0.439776	410	396	806	1398
MG-vs-FG	ko00982	Drug metabolism - cytochrome P450	0.02557	0.439776	8	13	21	28
MG-vs-FG	ko04512	ECM-receptor interaction	0.02705	0.443144	10	54	64	98
MG-vs-FG	ko00983	Drug metabolism - other enzymes	0.02923	0.45699	18	21	39	57
MG-vs-FG	ko05161	Hepatitis B	0.03445	0.490631	49	50	99	158
MG-vs-FG	ko00564	Glycerophospholipid metabolism	0.03473	0.490631	21	52	73	114
MG-vs-FG	ko05214	Glioma	0.03617	0.490631	30	28	58	89
MG-vs-FG	ko05034	Alcoholism	0.03708	0.490631	58	49	107	172
MG-vs-FG	ko00920	Sulfur metabolism	0.0397	0.492077	2	6	8	9

MG-vs-FG	ko03460	Fanconi anemia pathway	0.04108	0.492077	27	9	36	53
MG-vs-FG	ko05219	Bladder cancer	0.04355	0.492077	22	9	31	45
MG-vs-FG	ko00450	Selenocompound metabolism	0.04406	0.492077	8	4	12	15
MG-vs-FG	ko05215	Prostate cancer	0.04489	0.492077	34	49	83	132
MG-vs-FG	ko05202	Transcriptional misregulation in cancers	0.04577	0.492077	50	75	125	204
MG-vs-FG	ko00140	Steroid hormone biosynthesis	0.04819	0.502337	6	23	29	42
MG-vs-PMG	ko00524	Neomycin, kanamycin and gentamicin biosynthesis	0.00599	0.709956	1	1	2	7
MG-vs-PMG	ko04930	Type II diabetes mellitus	0.00733	0.709956	2	3	5	70
MG-vs-PMG	ko04910	Insulin signaling pathway	0.01095	0.709956	6	2	8	174
MG-vs-PMG	ko04120	Ubiquitin mediated proteolysis	0.01792	0.709956	6	1	7	154
MG-vs-PMG	ko04341	Hedgehog signaling pathway - fly	0.01934	0.709956	2	1	3	33
MG-vs-PMG	ko05210	Colorectal cancer	0.02033	0.709956	4	2	6	123
MG-vs-PMG	ko05412	rrhythmogenic right ventricular cardiomyopathy (ARV(	0.02107	0.709956	4	2	6	124
MG-vs-PMG	ko04520	Adherens junction	0.02946	0.709956	4	2	6	134
MG-vs-PMG	ko04064	NF-kappa B signaling pathway	0.03374	0.709956	2	3	5	103
MG-vs-PMG	ko05212	Pancreatic cancer	0.03497	0.709956	2	3	5	104
MG-vs-PMG	ko05221	Acute myeloid leukemia	0.04514	0.709956	3	1	4	77
MG-vs-PMG	ko04392	Hippo signaling pathway - multiple species	0.04677	0.709956	1	1	2	20
MG-vs-PMG	ko05213	Endometrial cancer	0.04882	0.709956	4	0	4	79
PMG-vs-FG	ko04110	Cell cycle	3.14E-06	0.001079	75	32	107	145
PMG-vs-FG	ko05322	Systemic lupus erythematosus	0.00018	0.030692	30	21	51	66
PMG-vs-FG	ko00240	Pyrimidine metabolism	0.00135	0.154898	47	26	73	104
PMG-vs-FG	ko00590	Arachidonic acid metabolism	0.00218	0.186705	10	24	34	44
PMG-vs-FG	ko00340	Histidine metabolism	0.00347	0.206172	11	7	18	21
PMG-vs-FG	ko04610	Complement and coagulation cascades	0.00361	0.206172	2	38	40	54
PMG-vs-FG	ko05161	Hepatitis B	0.00466	0.228116	51	53	104	158
PMG-vs-FG	ko04115	p53 signaling pathway	0.00962	0.379703	38	22	60	88
PMG-vs-FG	ko00260	Glycine, serine and threonine metabolism	0.01096	0.379703	17	14	31	42
PMG-vs-FG	ko05216	Thyroid cancer	0.01191	0.379703	17	19	36	50
PMG-vs-FG	ko00053	Ascorbate and aldarate metabolism	0.014	0.379703	8	11	19	24

PMG-vs-FG	ko05215	Prostate cancer	0.01401	0.379703	35	51	86	132
PMG-vs-FG	ko04218	Cellular senescence	0.01439	0.379703	63	77	140	223
PMG-vs-FG	ko04512	ECM-receptor interaction	0.01779	0.435939	10	55	65	98
PMG-vs-FG	ko03320	PPAR signaling pathway	0.02267	0.460502	19	34	53	79
PMG-vs-FG	ko03030	DNA replication	0.02335	0.460502	24	1	25	34
PMG-vs-FG	ko05222	Small cell lung cancer	0.02654	0.460502	37	38	75	116
PMG-vs-FG	ko00982	Drug metabolism - cytochrome P450	0.02665	0.460502	7	14	21	28
PMG-vs-FG	ko05206	MicroRNAs in cancer	0.02727	0.460502	62	72	134	216
PMG-vs-FG	ko00330	Arginine and proline metabolism	0.02933	0.460502	20	16	36	52
PMG-vs-FG	ko00983	Drug metabolism - other enzymes	0.03098	0.460502	17	22	39	57
PMG-vs-FG	ko05221	Acute myeloid leukemia	0.03463	0.460502	23	28	51	77
PMG-vs-FG	ko04979	Cholesterol metabolism	0.03559	0.460502	15	25	40	59
PMG-vs-FG	ko05202	Transcriptional misregulation in cancers	0.03735	0.460502	51	75	126	204
PMG-vs-FG	ko00564	Glycerophospholipid metabolism	0.03764	0.460502	22	51	73	114
PMG-vs-FG	ko01100	Metabolic pathways	0.03852	0.460502	404	401	805	1398
PMG-vs-FG	ko03013	RNA transport	0.03856	0.460502	65	41	106	170
PMG-vs-FG	ko04640	Hematopoietic cell lineage	0.03875	0.460502	12	43	55	84
PMG-vs-FG	ko04974	Protein digestion and absorption	0.03893	0.460502	15	68	83	131
PMG-vs-FG	ko00920	Sulfur metabolism	0.04057	0.463865	2	6	8	9
PMG-vs-FG	ko05219	Bladder cancer	0.0457	0.490127	21	10	31	45
PMG-vs-FG	ko04662	B cell receptor signaling pathway	0.04697	0.490127	11	40	51	78
PMG-vs-FG	ko03440	Homologous recombination	0.04716	0.490127	22	4	26	37
MB-vs-FB	ko04114	Oocyte meiosis	0.00141	0.316191	8	0	8	126
MB-vs-FB	ko05130	Pathogenic Escherichia coli infection	0.00437	0.36709	3	5	8	151
MB-vs-FB	ko04120	Ubiquitin mediated proteolysis	0.00492	0.36709	7	1	8	154
MB-vs-FB	ko04110	Cell cycle	0.01226	0.579657	6	1	7	145
MB-vs-FB	ko04210	Apoptosis	0.01294	0.579657	5	3	8	182
MB-vs-FB	ko00520	Amino sugar and nucleotide sugar metabolism	0.02274	0.848985	3	1	4	63
MB-vs-FB	ko00130	Ubiquinone and other terpenoid-quinone biosynthesis	0.03005	0.888856	2	0	2	16
MB-vs-FB	ko03420	Nucleotide excision repair	0.0329	0.888856	0	3	3	41

MB-vs-FB	ko00250	Alanine, aspartate and glutamate metabolism	0.03939	0.888856	3	0	3	44
MB-vs-FB	ko05012	Parkinson disease	0.0442	0.888856	5	1	6	150
MB-vs-PMB	ko04120	Ubiquitin mediated proteolysis	0.003	0.303667	6	1	7	154
MB-vs-PMB	ko00524	Neomycin, kanamycin and gentamicin biosynthesis	0.0031	0.303667	1	1	2	7
MB-vs-PMB	ko05012	Parkinson disease	0.01086	0.567522	5	1	6	150
MB-vs-PMB	ko03420	Nucleotide excision repair	0.0142	0.567522	0	3	3	41
MB-vs-PMB	ko05010	Alzheimer disease	0.01448	0.567522	6	1	7	207
MB-vs-PMB	ko00360	Phenylalanine metabolism	0.01851	0.604556	2	0	2	17
MB-vs-PMB	ko03018	RNA degradation	0.02383	0.667234	3	1	4	88
MB-vs-PMB	ko01200	Carbon metabolism	0.02863	0.701375	2	3	5	138
MB-vs-PMB	ko00520	Amino sugar and nucleotide sugar metabolism	0.04353	0.933393	2	1	3	63
PMB-vs-FB	ko05130	Pathogenic Escherichia coli infection	0.00095	0.083036	1	4	5	151
PMB-vs-FB	ko00232	Caffeine metabolism	0.01527	0.463018	0	1	1	3
PMB-vs-FB	ko02010	ABC transporters	0.01597	0.463018	0	2	2	38
PMB-vs-FB	ko04540	Gap junction	0.02405	0.465862	0	3	3	122
PMB-vs-FB	ko05216	Thyroid cancer	0.02677	0.465862	1	1	2	50
PMB-vs-FB	ko04145	Phagosome	0.04421	0.641048	0	3	3	155
ML-vs-FL	ko00910	Nitrogen metabolism	0.0087	0.670971	2	1	3	23
ML-vs-FL	ko04972	Pancreatic secretion	0.016	0.670971	4	3	7	140
ML-vs-FL	ko04110	Cell cycle	0.01905	0.670971	6	1	7	145
ML-vs-FL	ko00030	Pentose phosphate pathway	0.02339	0.670971	2	1	3	33
ML-vs-FL	ko04120	Ubiquitin mediated proteolysis	0.02553	0.670971	7	0	7	154
ML-vs-FL	ko05214	Glioma	0.02557	0.670971	2	3	5	89
ML-vs-FL	ko05225	Hepatocellular carcinoma	0.03003	0.670971	4	4	8	195
ML-vs-FL	ko00520	Amino sugar and nucleotide sugar metabolism	0.03014	0.670971	3	1	4	63
ML-vs-FL	ko04114	Oocyte meiosis	0.03085	0.670971	6	0	6	126
ML-vs-FL	ko05224	Breast cancer	0.03247	0.670971	4	4	8	198
ML-vs-FL	ko04914	Progesterone-mediated oocyte maturation	0.03269	0.670971	5	0	5	95
ML-vs-FL	ko04142	Lysosome	0.03531	0.670971	5	2	7	165
ML-vs-FL	ko05220	Chronic myeloid leukemia	0.04548	0.713475	2	3	5	104

ML-vs-FL	ko00730	Thiamine metabolism	0.04852	0.713475	1	1	2	19
ML-vs-FL	ko00250	Alanine, aspartate and glutamate metabolism	0.04897	0.713475	3	0	3	44
ML-vs-PML	ko04972	Pancreatic secretion	0.00029	0.063766	4	4	8	140
ML-vs-PML	ko00591	Linoleic acid metabolism	0.00106	0.092116	1	2	3	17
ML-vs-PML	ko00592	alpha-Linolenic acid metabolism	0.00126	0.092116	1	2	3	18
ML-vs-PML	ko00010	Glycolysis / Gluconeogenesis	0.00227	0.109124	0	5	5	76
ML-vs-PML	ko04975	Fat digestion and absorption	0.00248	0.109124	1	3	4	47
ML-vs-PML	ko05012	Parkinson disease	0.00983	0.310524	5	1	6	150
ML-vs-PML	ko04120	Ubiquitin mediated proteolysis	0.01111	0.310524	6	0	6	154
ML-vs-PML	ko00564	Glycerophospholipid metabolism	0.01254	0.310524	3	2	5	114
ML-vs-PML	ko00561	Glycerolipid metabolism	0.01623	0.310524	2	2	4	80
ML-vs-PML	ko00250	Alanine, aspartate and glutamate metabolism	0.01623	0.310524	3	0	3	44
ML-vs-PML	ko00590	Arachidonic acid metabolism	0.01623	0.310524	1	2	3	44
ML-vs-PML	ko04270	Vascular smooth muscle contraction	0.01694	0.310524	4	2	6	169
ML-vs-PML	ko00565	Ether lipid metabolism	0.02162	0.365825	1	2	3	49
ML-vs-PML	ko01100	Metabolic pathways	0.02626	0.387432	12	13	25	1398
ML-vs-PML	ko01200	Carbon metabolism	0.02642	0.387432	1	4	5	138
ML-vs-PML	ko00910	Nitrogen metabolism	0.03155	0.43379	1	1	2	23
ML-vs-PML	ko00520	Amino sugar and nucleotide sugar metabolism	0.04128	0.534204	2	1	3	63
PML-vs-FL	ko03320	PPAR signaling pathway	0.0001	0.014376	2	3	5	79
PML-vs-FL	ko00500	Starch and sucrose metabolism	0.00128	0.088775	2	1	3	36
PML-vs-FL	ko00730	Thiamine metabolism	0.00571	0.264412	1	1	2	19
PML-vs-FL	ko01100	Metabolic pathways	0.01427	0.391252	9	6	15	1398
PML-vs-FL	ko04115	p53 signaling pathway	0.01579	0.391252	1	2	3	88
PML-vs-FL	ko00790	Folate biosynthesis	0.01768	0.391252	1	1	2	34
PML-vs-FL	ko00052	Galactose metabolism	0.0197	0.391252	2	0	2	36
PML-vs-FL	ko00250	Alanine, aspartate and glutamate metabolism	0.0287	0.400739	1	1	2	44
PML-vs-FL	ko05162	Measles	0.029	0.400739	0	3	3	111
PML-vs-FL	ko05222	Small cell lung cancer	0.03245	0.400739	2	1	3	116
PML-vs-FL	ko01212	Fatty acid metabolism	0.03767	0.400739	2	0	2	51

PML-vs-FL	ko04973	Carbohydrate digestion and absorption	0.03903	0.400739	2	0	2	52
PML-vs-FL	ko05206	MicroRNAs in cancer	0.04049	0.400739	2	2	4	216
PML-vs-FL	ko00524	Neomycin, kanamycin and gentamicin biosynthesis	0.0415	0.400739	1	0	1	7
PML-vs-FL	ko04974	Protein digestion and absorption	0.04406	0.400739	3	0	3	131
PML-vs-FL	ko04340	Hedgehog signaling pathway	0.04905	0.400739	1	1	2	59
PML-vs-FL	ko04979	Cholesterol metabolism	0.04905	0.400739	1	1	2	59
MM-vs-FM	ko04110	Cell cycle	1.59E-17	5.02E-15	52	1	53	145
MM-vs-FM	ko05322	Systemic lupus erythematosus	7.01E-08	1.10E-05	19	4	23	66
MM-vs-FM	ko03030	DNA replication	4.00E-07	4.20E-05	15	0	15	34
MM-vs-FM	ko03460	Fanconi anemia pathway	2.87E-06	2.26E-04	18	0	18	53
MM-vs-FM	ko04914	Progesterone-mediated oocyte maturation	7.34E-06	4.42E-04	25	0	25	95
MM-vs-FM	ko04114	Oocyte meiosis	8.42E-06	4.42E-04	27	3	30	126
MM-vs-FM	ko04218	Cellular senescence	1.52E-05	6.85E-04	29	15	44	223
MM-vs-FM	ko03440	Homologous recombination	4.62E-05	1.82E-03	13	0	13	37
MM-vs-FM	ko03430	Mismatch repair	0.00074	2.23E-02	8	0	8	21
MM-vs-FM	ko03040	Spliceosome	0.00076	2.23E-02	25	1	26	131
MM-vs-FM	ko00240	Pyrimidine metabolism	0.00078	2.23E-02	19	3	22	104
MM-vs-FM	ko05034	Alcoholism	0.00135	3.55E-02	28	3	31	172
MM-vs-FM	ko03018	RNA degradation	0.00334	8.09E-02	18	0	18	88
MM-vs-FM	ko00061	Fatty acid biosynthesis	0.01047	2.36E-01	4	1	5	14
MM-vs-FM	ko05216	Thyroid cancer	0.01149	2.41E-01	7	4	11	50
MM-vs-FM	ko05213	Endometrial cancer	0.01402	2.68E-01	11	4	15	79
MM-vs-FM	ko04120	Ubiquitin mediated proteolysis	0.01444	2.68E-01	23	2	25	154
MM-vs-FM	ko04115	p53 signaling pathway	0.01699	2.97E-01	16	0	16	88
MM-vs-FM	ko00982	Drug metabolism - cytochrome P450	0.02081	3.35E-01	4	3	7	28
MM-vs-FM	ko03420	Nucleotide excision repair	0.02159	3.35E-01	9	0	9	41
MM-vs-FM	ko00230	Purine metabolism	0.02231	3.35E-01	28	4	32	216
MM-vs-FM	ko03013	RNA transport	0.0259	3.71E-01	22	4	26	170
MM-vs-FM	ko03022	Basal transcription factors	0.03712	5.08E-01	8	0	8	38
MM-vs-FM	ko04540	Gap junction	0.04404	5.49E-01	12	7	19	122

MM-vs-FM	ko05168	Herpes simplex infection	0.04475	5.49E-01	24	3	27	187
MM-vs-FM	ko05210	Colorectal cancer	0.04727	5.49E-01	15	4	19	123
MM-vs-FM	ko03410	Base excision repair	0.04791	5.49E-01	7	0	7	33
MM-vs-PMM	ko04614	Renin-angiotensin system	0.00221	0.263915	2	1	3	19
MM-vs-PMM	ko04270	Vascular smooth muscle contraction	0.0025	0.263915	6	2	8	169
MM-vs-PMM	ko04120	Ubiquitin mediated proteolysis	0.00579	0.336218	6	1	7	154
MM-vs-PMM	ko05010	Alzheimer disease	0.00845	0.336218	7	1	8	207
MM-vs-PMM	ko04022	cGMP - PKG signaling pathway	0.0094	0.336218	8	1	9	255
MM-vs-PMM	ko04974	Protein digestion and absorption	0.01009	0.336218	6	0	6	131
MM-vs-PMM	ko04512	ECM-receptor interaction	0.01201	0.336218	4	1	5	98
MM-vs-PMM	ko04510	Focal adhesion	0.01275	0.336218	6	3	9	268
MM-vs-PMM	ko04141	Protein processing in endoplasmic reticulum	0.01464	0.343235	4	3	7	184
MM-vs-PMM	ko05012	Parkinson disease	0.01866	0.393751	5	1	6	150
MM-vs-PMM	ko04520	Adherens junction	0.03988	0.762802	4	1	5	134
MM-vs-PMM	ko04924	Renin secretion	0.04711	0.762802	3	1	4	97
MM-vs-PMM	ko04340	Hedgehog signaling pathway	0.04963	0.762802	1	2	3	59
PMM-vs-FM	ko04110	Cell cycle	1.54E-19	4.72E-17	49	0	49	145
PMM-vs-FM	ko03030	DNA replication	1.10E-08	1.69E-06	15	0	15	34
PMM-vs-FM	ko05322	Systemic lupus erythematosus	8.34E-08	8.54E-06	17	3	20	66
PMM-vs-FM	ko03440	Homologous recombination	8.68E-05	6.53E-03	11	0	11	37
PMM-vs-FM	ko04114	Oocyte meiosis	0.00011	6.53E-03	22	1	23	126
PMM-vs-FM	ko04914	Progesterone-mediated oocyte maturation	0.00013	6.53E-03	19	0	19	95
PMM-vs-FM	ko00240	Pyrimidine metabolism	0.00015	6.61E-03	17	3	20	104
PMM-vs-FM	ko04115	p53 signaling pathway	0.00044	1.68E-02	16	1	17	88
PMM-vs-FM	ko03430	Mismatch repair	0.00081	2.75E-02	7	0	7	21
PMM-vs-FM	ko04218	Cellular senescence	0.00263	7.74E-02	25	5	30	223
PMM-vs-FM	ko04964	Proximal tubule bicarbonate reclamation	0.00277	7.74E-02	7	2	9	39
PMM-vs-FM	ko05206	MicroRNAs in cancer	0.00317	8.10E-02	26	3	29	216
PMM-vs-FM	ko00512	Mucin type O-glycan biosynthesis	0.00757	1.57E-01	7	0	7	30
PMM-vs-FM	ko05219	Bladder cancer	0.00759	1.57E-01	8	1	9	45

PMM-vs-FM	ko03460	Fanconi anemia pathway	0.00766	1.57E-01	10	0	10	53
PMM-vs-FM	ko03018	RNA degradation	0.00861	1.65E-01	14	0	14	88
PMM-vs-FM	ko03420	Nucleotide excision repair	0.01342	2.42E-01	8	0	8	41
PMM-vs-FM	ko05034	Alcoholism	0.01618	2.76E-01	20	2	22	172
PMM-vs-FM	ko01524	Platinum drug resistance	0.02233	3.61E-01	10	0	10	62
PMM-vs-FM	ko00910	Nitrogen metabolism	0.03085	4.73E-01	4	1	5	23
PMM-vs-FM	ko00230	Purine metabolism	0.03348	4.89E-01	21	4	25	216
PMM-vs-FM	ko05216	Thyroid cancer	0.04053	5.59E-01	6	2	8	50
PMM-vs-FM	ko03410	Base excision repair	0.04191	5.59E-01	6	0	6	33
PMM-vs-FM	ko00601	lycosphingolipid biosynthesis - lacto and neolacto seri	0.04946	6.15E-01	7	0	7	43

Table S3. The sample growth traits used in WGCNA.

Sample	Sample Body length (Average)	Body length for three individuals	Sample Body width (Average)	Body width for three individuals	Sample Body weight (Average)	Body weight for three individuals
FB1	51.00	47.00; 54.00; 52.00	15.50	16.00; 15.50; 15.00	857.27	917.00; 846.10; 808.70
FB2	52.17	53.00; 50.50; 53.00	15.00	16.00; 14.00; 15.00	775.33	903.00; 727.00; 696.00
FB3	51.00	49.00; 54.00; 50.00	14.17	14.00; 15.00; 13.50	770.33	798.00; 823.00; 690.00
FG1	51.00	47.00; 54.00; 52.00	15.50	16.00; 15.50; 15.00	857.27	917.00; 846.10; 808.70
FG2	52.17	53.00; 50.50; 53.00	15.00	16.00; 14.00; 15.00	775.33	903.00; 727.00; 696.00
FG3	51.00	49.00; 54.00; 50.00	14.17	14.00; 15.00; 13.50	770.33	798.00; 823.00; 690.00
FL1	51.00	47.00; 54.00; 52.00	15.50	16.00; 15.50; 15.00	857.27	917.00; 846.10; 808.70
FL2	52.17	53.00; 50.50; 53.00	15.00	16.00; 14.00; 15.00	775.33	903.00; 727.00; 696.00
FL3	51.00	49.00; 54.00; 50.00	14.17	14.00; 15.00; 13.50	770.33	798.00; 823.00; 690.00
FM1	51.00	47.00; 54.00; 52.00	15.50	16.00; 15.50; 15.00	857.27	917.00; 846.10; 808.70
FM2	52.17	53.00; 50.50; 53.00	15.00	16.00; 14.00; 15.00	775.33	903.00; 727.00; 696.00
FM3	51.00	49.00; 54.00; 50.00	14.17	14.00; 15.00; 13.50	770.33	798.00; 823.00; 690.00
MB1	31.33	31.00; 32.00, 31.00	8.50	8.00; 9.00; 8.50	176.83	169.00; 195.80; 165.70
MB2	31.17	31.00; 32.00; 30.50	8.83	9.00; 8.50; 9.00	159.73	151.70; 161.30; 166.20
MB3	30.67	31.00; 30.50; 30.50	7.67	8.00; 7.50; 7.50	135.60	138.20; 141.00; 127.60
MG1	31.33	31.00; 32.00, 31.00	8.50	8.00; 9.00; 8.50	176.83	169.00; 195.80; 165.70
MG2	31.17	31.00; 32.00; 30.50	8.83	9.00; 8.50; 9.00	159.73	151.70; 161.30; 166.20
MG3	30.67	31.00; 30.50; 30.50	7.67	8.00; 7.50; 7.50	135.60	138.20; 141.00; 127.60
ML1	31.33	31.00; 32.00, 31.00	8.50	8.00; 9.00; 8.50	176.83	169.00; 195.80; 165.70
ML2	31.17	31.00; 32.00; 30.50	8.83	9.00; 8.50; 9.00	159.73	151.70; 161.30; 166.20
ML3	30.67	31.00; 30.50; 30.50	7.67	8.00; 7.50; 7.50	135.60	138.20; 141.00; 127.60
MM1	31.33	31.00; 32.00, 31.00	8.50	8.00; 9.00; 8.50	176.83	169.00; 195.80; 165.70
MM2	31.17	31.00; 32.00; 30.50	8.83	9.00; 8.50; 9.00	159.73	151.70; 161.30; 166.20
MM3	30.67	31.00; 30.50; 30.50	7.67	8.00; 7.50; 7.50	135.60	138.20; 141.00; 127.60
PMB2	32.50	33.00; 33.00; 31.50	8.00	8.00; 8.00; 8.00	167.47	164.10; 175.80; 162.50
PMB3	30.33	28.00; 33.00; 30.00	7.83	7.50; 8.00; 8.00	167.87	145.20; 169.60; 188.80

PMG1	32.00	31.00; 33.00; 32.00	8.00	7.50; 8.50; 8.00	170.73	145.30; 200.00; 166.90
PMG2	32.50	33.00; 33.00; 31.50	8.00	8.00; 8.00; 8.00	167.47	164.10; 175.80; 162.50
PMG3	30.33	28.00; 33.00; 30.00	7.83	7.50; 8.00; 8.00	167.87	145.20; 169.60; 188.80
PML1	32.00	31.00; 33.00; 32.00	8.00	7.50; 8.50; 8.00	170.73	145.30; 200.00; 166.90
PML2	32.50	33.00; 33.00; 31.50	8.00	8.00; 8.00; 8.00	167.47	164.10; 175.80; 162.50
PML3	30.33	28.00; 33.00; 30.00	7.83	7.50; 8.00; 8.00	167.87	145.20; 169.60; 188.80
PMM1	32.00	31.00; 33.00; 32.00	8.00	7.50; 8.50; 8.00	170.73	145.30; 200.00; 166.90
PMM2	32.50	33.00; 33.00; 31.50	8.00	8.00; 8.00; 8.00	167.47	164.10; 175.80; 162.50
PMM3	30.33	28.00; 33.00; 30.00	7.83	7.50; 8.00; 8.00	167.87	145.20; 169.60; 188.80

Table S4. The primers used in present study.

Tissue	Gene ID	Gene Symbol	name	primer sequences
brain	103396969	growth arrest and DNA damage-inducible protein GADD45 gamma	gadd45g	GCTGATGATGGTGAAAAG
				AAATCCCTCAAAAAGTGA
brain	MSTRG.27059	Ubiquitin-conjugating enzyme E2L3	ube2l3	TGTTTTGTCGTTTTGATT
				AGGGTCTTTGACTGCTAT
liver (gonad)	103396100	Cyclin Dependent Kinase 7	cdc7	CCTTCAGTTCAGTTTACC
				CATCACATTCTCTTTGCC
liver	103386169	cell death activator CIDE-3	cidec	AAGAACAACCCCAAGGAT
				ATGTAGCAAGAGGAGCCC
gonad	103389623	Origin recognition complex subunit 4	orc4	GTTTGACCTCTTCGCCCA
				GACTTCACCCGCTTCTCC
gonad	103397789	proliferating cell nuclear antigen	pcna	ACGAGGACATCATCACCC
				GCATTTTCACCACACAGC
gonad	103390614	minichromosome maintenance proteins 7	mcm7	GGGGTCGTCTCTACTTGC
				TTGACTGAATCCTGCTTT
gonad	103395116	yorkie homolog	yap1	GTTTACCCCGCACATC
				GTCGCCCTCAGACATT
gonad	103382170	bone morphogenetic protein 2	bmp2	GAGGAGCAGGAACCACCA
				ATCTTCGGCAGCACAACT
muscle	103397325	Ubiquitin C	ubc	AGAGGAAGGATTTGAGCG
				AGGTGTGATGTCGTAGGC
muscle	103382745	Origin recognition complex subunit 5	orc5	AAGAAAAACGAAAAGACG
				GAAATGGCACAGATGAAG
muscle	103389033	cyclin B1	ccnb1	GCCATCATTGACCGCTTT
				CCGACCCAGTTGGAACTT
all tissues	103393304	beta actin	β-actin	TTCCAGCCTTCCTTCCTT
				TACCTCCAGACAGCACAG

Table S5. The sequening data information in the WGBS.

Sample	Clean Reads Num	HQ Clean Reads Num(%)	Read Length	Adapter(%)	Low Quality(%)	N(%)	Mapped Ratio(%)	Sequence Depth
FG1	111029356	110542962 (99.56%)	150/150	692 (0%)	485366 (0.44%)	336 (0.0%)	70.2	26.15
FG2	112306940	111748082 (99.5%)	150/150	494 (0%)	557990 (0.5%)	374 (0.0%)	69.91	26.32
FG3	113147380	112596848 (99.51%)	150/150	588 (0%)	549566 (0.49%)	378 (0.0%)	70.39	26.7
FM1	113706226	113209722 (99.56%)	150/150	566 (0%)	489316 (0.43%)	6622 (0.01%)	69.53	26.52
FM2	112313732	111844468 (99.58%)	150/150	700 (0%)	462766 (0.41%)	5798 (0.01%)	70.34	26.51
FM3	110305108	109727720 (99.48%)	150/150	476 (0%)	576504 (0.52%)	408 (0.0%)	70.14	25.93
MG1	110480040	110020972 (99.58%)	150/150	550 (0%)	458118 (0.41%)	400 (0.0%)	71.28	26.43
MG2	109414182	109035938 (99.65%)	150/150	410 (0%)	371380 (0.34%)	6454 (0.01%)	71.78	26.37
MG3	106360760	105945058 (99.61%)	150/150	484 (0%)	409094 (0.38%)	6124 (0.01%)	71.06	25.37
MM1	107172150	106750238 (99.61%)	150/150	542 (0%)	415064 (0.39%)	6306 (0.01%)	70.67	25.42
MM2	154243232	153305570 (99.39%)	150/150	1330 (0%)	935892 (0.61%)	440 (0.0%)	69.39	35.85
MM3	115589994	115113764 (99.59%)	150/150	624 (0%)	468978 (0.41%)	6628 (0.01%)	70.75	27.44
PMG1	156672578	155868440 (99.49%)	150/150	1326 (0%)	802374 (0.51%)	438 (0.0%)	70.25	36.9
PMG2	109047052	108503390 (99.5%)	150/150	520 (0%)	542820 (0.5%)	322 (0.0%)	70.43	25.75
PMG3	116577536	116040944 (99.54%)	150/150	434 (0%)	535750 (0.46%)	408 (0.0%)	70.6	27.6
PMM1	109496918	108933954 (99.49%)	150/150	508 (0%)	562082 (0.51%)	374 (0.0%)	69.79	25.62
PMM2	110362622	109618918 (99.33%)	150/150	1176 (0%)	736050 (0.67%)	6478 (0.01%)	69.13	25.53
PMM3	112593252	111843020 (99.33%)	150/150	1498 (0%)	742150 (0.66%)	6584 (0.01%)	68.98	26

Table S6. The genomic DNA methylation levels in 18 samples.

Sample	C(%)	CG(%)	CHG(%)	CHH(%)
FG1	7.27	65.48	0.74	0.78
FG2	7.27	66.02	0.68	0.71
FG3	7.31	66.5	0.68	0.71
FM1	7.29	65.87	0.68	0.75
FM2	7.27	65.88	0.68	0.74
FM3	7.24	65.39	0.7	0.77
MG1	8.55	80.99	0.45	0.47
MG2	8.48	80.5	0.46	0.48
MG3	8.78	80.89	0.69	0.72
MM1	7.42	67.34	0.68	0.73
MM2	7.27	68.1	0.46	0.51
MM3	7.59	68.69	0.72	0.77
PMG1	8.67	80.28	0.65	0.68
PMG2	8.87	81.53	0.73	0.75
PMG3	8.8	81	0.71	0.74
PMM1	7.8	71.25	0.71	0.76
PMM2	7.4	67.14	0.73	0.79
PMM3	7.68	69.98	0.73	0.79
